# Tandem domain structure determination based on a systematic enumeration of conformations

**DOI:** 10.1038/s41598-021-96370-z

**Published:** 2021-08-19

**Authors:** Thérèse E. Malliavin

**Affiliations:** 1 Unité de Bioinformatique Structurale, Institut Pasteur, UMR 3528, CNRS, Paris, France; 2 Center of Bioinformatics, Biostatistics and Integrative Biology, Institut Pasteur, USR 3756, CNRS, Paris, France

**Keywords:** Computational biophysics, Molecular modelling, Computational science, Solution-state NMR

## Abstract

Protein structure determination is undergoing a change of perspective due to the larger importance taken in biology by the disordered regions of biomolecules. In such cases, the convergence criterion is more difficult to set up and the size of the conformational space is a obstacle to exhaustive exploration. A pipeline is proposed here to exhaustively sample protein conformations using backbone angle limits obtained by nuclear magnetic resonance (NMR), and then to determine the populations of conformations. The pipeline is applied to a tandem domain of the protein whirlin. An original approach, derived from a reformulation of the Distance Geometry Problem is used to enumerate the conformations of the linker connecting the two domains. Specifically designed procedure then permit to assemble the domains to the linker conformations and to optimize the tandem domain conformations with respect to two sets of NMR measurements: residual dipolar couplings and paramagnetic resonance enhancements. The relative populations of optimized conformations are finally determined by fitting small angle X-ray scattering (SAXS) data. The most populated conformation of the tandem domain is a semi-closed one, fully closed and more extended conformations being in minority, in agreement with previous observations. The SAXS and NMR data show different influences on the determination of populations.

## Introduction

During the last two decades, interest in the involvement of disordered regions of proteins in biological processes has steadily increased^1^. The influence of disordered regions of proteins has been demonstrated in many phenomena such as: moonlighting in the network of interactions between proteins^2^ or in post-translational modifications^3^, short linear patterns (SLiMS)^4,5^ and molecular recognition functionalities (MoRF)^6^. In addition, proteins containing intrinsically disordered regions tend to act as hubs in protein interactome^7^.

The study of disordered regions of proteins is made difficult because most of biophysical techniques concentrates on time-average or space-average data in order to obtain sufficient signal-to-noise ratio. In that frame, the observation of disordered molecules is hampered by the destruction of a part of the observable signal by averaging on heterogeneous sets of conformations. On the other hand, the signal produced by individual conformations is in most of the cases not strong enough to be recorded. Although nuclear magnetic resonance (NMR) is a method of choice for studying conformations of disordered regions of proteins, it faces problems similar to those of other biophysical approaches. Consequently, the approaches traditionally adopted to determine the structural set of proteins for disordered regions rely on two steps: firstly, the generation of a set of conformations, secondly the fitting of these conformations to various biophysical measurements to determine their relative populations. The conformations are classically generated from databases built in advance from the statistical analysis of protein structures^8–10^. Here, we propose a new point of view where all possible conformations are systematically enumerated within limits determined from measurements of chemical shifts.

This enumeration problem is addressed as an interval branch-and-prune (iBP) problem^11–16^ in which the protein structure is described as a tree and the available experimental measurements permit tree branching and pruning. As the iBP description makes possible a discrete enumeration of all possible solutions, it strongly contrasts with most of the optimization approaches used for biomolecular structure calculations, as for example the simulated annealing procedure^17^. The approach iBP rejoins the work of Feldman and Hogue^18^ who analyzed the exploration of the conformational space of proteins by systematic enumeration. The threading-augmented interval Branch-and-Prune (TAiBP) approach was recently proposed^19,20^ as a reformulation of iBP approach^11^. TAiBP provides a framework for the systematic enumeration of protein conformations, while allowing overcome the combinatorial barrier intrinsic to this enumeration, and will thus be used here for enumerating conformations in disordered protein regions.

Numerous proteins involved in cell signaling are formed of several folded domain connected by more or less disordered linkers. Such proteins, the so-called tandem domain proteins, play important role in various physiological processes^21–25^. The folded domains have usually well-known structural properties whereas the conformations of disordered linkers are less determined, but play a key role to understand the conformational landscapes of tandem domain proteins and thus their functions.

**Figure 1:**
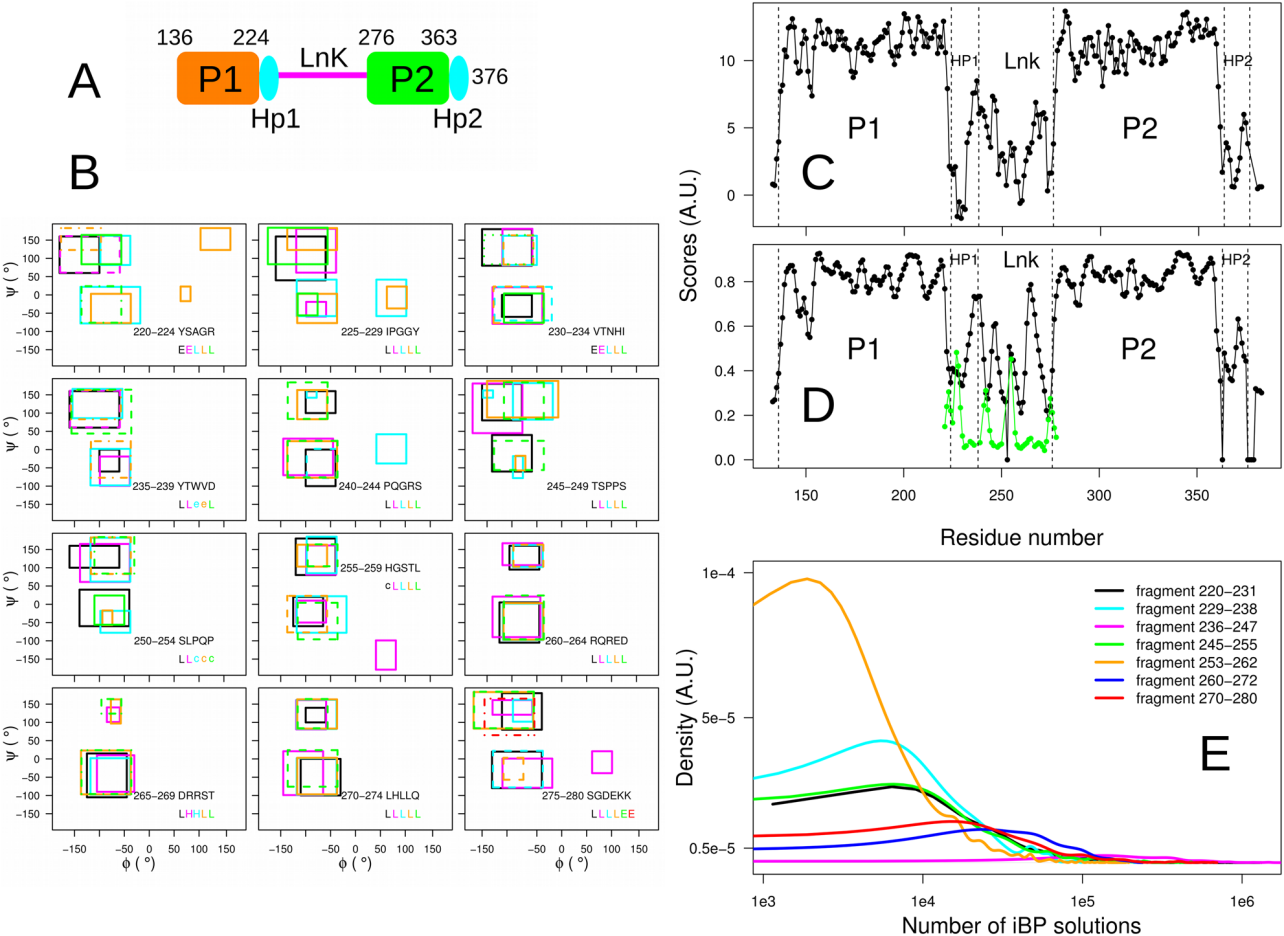
Exploration of the conformational space of Lnk. (**A**) Scheme of the primary structure of the tandem domain protein P1LnkP2 spanning residues 136–376 of protein whirlin. The $\beta$ hairpins located after the PDZ domains P1 and P2 and corresponding to residues 225–238 and 363–376 are colored in cyan and labeled Hp1 and Hp2. (**B**) Boxes defining the $\phi$ and $\psi$ intervals sampled during the iBP calculations. On each panel, for five consecutive residues of the linker (six residues 275–280 on the right bottom panel), the boxes are drawn in different colors for each residue and are plotted with different line types in order to reduce box overlays. The corresponding residue sequences are given on each panel along with the secondary structure prediction (H,E,L,e,c) proposed by TALOS-N^28^, colored in the same way than the boxes. C-D. Disorder prediction along the residues of P1LnkP2 by Chemical shift Z-score for assessing Order/Disorder (CHEZOD)^29^ (**C**), by random coil index (RCI)^30^ calculated with TALOS-N^28^ (**D**, black curve) and by 1-coil (**D**, green curve) where coil is the percentage of coil predicted by $\delta$2D^31^. As the $\delta$2D approach is specially designed for disordered protein regions, it was only applied on the Lnk region. (**E**) Distribution of the number of solutions for individual iBP calculations realized on a given peptide fragment. For the sake of clarity, the two axes are drawn in logarithmic scale. The panel (**A**) was prepared using LibreOffice 6.0.7.3^57^ and the other panels using R 3.4.1^58^.

The approach for systematically exploring the conformational landscape of proteins focuses here is on the calculation of a tandem domain of the protein whirlin, a scaffold protein essential for the assembly of the USH2 protein complex^26^. A failure in the formation of this complex is observed in the Usher syndrome, a genetic disorder resulting in a combination of hearing loss and visual impairment. The tandem domain of whirlin spans the residues 136–376 and is formed of a PDZ domain, named hereafter P1 (residues 136–224), of a linker named hereafter Lnk (residues 225–280), and of a second PDZ domain (residues 281–362) followed by a $\beta$ hairpin (residues 363–376), and named hereafter P2 (Fig. 1A). The structure of P1LnkP2 has been previously extensively studied by various NMR approaches as well as SAXS measurements^27^, but this enormous effort did not produce an exhaustive description of the protein structure including Lnk conformations.

## Results

A several steps approach was here adopted for the calculations of P1LnkP2 conformations, including: (1) the use of nuclear magnetic resonance (NMR) chemical shifts measured on Lnk coupled to the TAiBP approach to systematically enumerate Lnk conformations; (2) the assembly of P1 and P2 folded structures to Lnk conformations; (3) the optimization of P1LnkP2 conformations with respect to measured NMR residual dipolar couplings (RDC) and paramagnetic resonance enhancements (PRE)^27^; (4) the determination of the populations of P1LnkP2 conformations using small angle X-ray scattering (SAXS) data.

### Extraction of $\phi$, $\psi$ boxes used for enumerating the Lnk conformations

Using the chemical shifts measured for the nuclei H$\alpha$, HN, $^{15}$N, $^{13}$C$\alpha$, $^{13}$C$\beta$ of Lnk residues, the output of TALOS-N^28^ was used to determine boxes of $\phi$ and $\psi$ values, giving the limits in which the conformations of Lnk will be enumerated. TALOS-N uses an artificial neural network (ANN) to predict the 324-state $\phi$/$\psi$ distribution of residue *i* on the basis of the NMR chemical shifts and of the residues adjacent to *i*. The 324-state $\phi$/$\psi$ distribution corresponds to the likelihood that residue *i* adopts torsion angles that fall in any of the 324 voxels, of 20$^{\circ }$ $\times$ 20$^{\circ }$ each, that make up the Ramachandran map^28^. Classically, as TALOS-N initially intends to predict a single region of $\phi$, $\psi$ values in order to build dihedral angle boxes used for optimization of protein structure, either the regions of the Ramachandran map with smallest likelihood have been classically assumed to correspond to statistically unlikely regions, either the $\phi$, $\psi$ angles were pointed as unpredictable in the case where several regions of the Ramachandran maps displayed comparable likelihood values. The present work proposes to take a new point of view in which all regions of Ramachandran map displaying likelihood larger than a given threshold may correspond to Ramachandran regions populated by some conformations present in solution for the studied protein. Using this point of view, a protocol is described to exhaustively enumerate all possible conformations of Lnk within the limits of the selected Ramachandran regions, as described in the Section “Enumeration of Lnk conformations” in Methods.

In order to obtain the $\phi$ and $\psi$ boxes which will be used for sampling protein conformations, each $\phi$/$\psi$ distribution produced by TALOS-N for a given residue was normalized in order that the sum of all voxels values is equal to one. Then, all voxels for which the normalized value was larger than 0.01, were selected. Boxes were manually chosen overlying selected voxels. As for the residue P254, no $\phi$/$\psi$ distribution could be determined by TALOS-N, the same boxes than the ones chosen for H255 were used for this residue. The $\phi$ and $\psi$ lower and upper limits defining the boxes are given in Table S1.

The boxes are drawn in the Ramachandran maps (Fig. 1B) for stretches of successive five or six residues in the linker. For most of the residues, two boxes have been extracted from the $\phi$/$\psi$ distributions, one being located in the negative $\phi$ and $\psi$ values close to the $\alpha$ helix, and the other being located in the negative $\phi$ and positive $\psi$ values close to the $\beta$ region. For few residues, a box was also detected in the region of positive $\phi$ values. The predictions of secondary structures produced by TALOS-N, and also given in the panels of Fig. 1B, are in agreement with the boxes. Indeed, the residues predicted to be in “E” structure display a box containing the $\beta$ strand region and the residues predicted to be in “H” structure display a box containing the $\alpha$ helix region. The residues predicted to be in the secondary structure “L” or “c” displays mostly at least two boxes located in the neighborhood of $\alpha$ helix or $\beta$ strand and for some residues, additional boxes located in the positive $\phi$ region. This observation agrees with a description of “L” or “c” secondary structures corresponding to an equilibrium between conformations with various secondary structures.

From the chemical shift values, relative order has been predicted along the P1LnkP2 residues using several approaches: the Chemical shift Z-score for assessing Order/Disorder (CHEZOD)^29^, the random coil index (RCI) score^30^ implemented in TALOS-N and the method $\delta$2D^31^ (Fig. 1C,D). CHEZOD (Fig. 1C) and RCI (Fig. 1D, black curve) display similar profiles. For both methods, Lnk is more disordered than P1 and P2, but some Lnk regions display order peaks. At the contrary, the plot of 1-coil, where coil represents the percentage of coil value determined by $\delta$2D^31^ (Fig. 1D, green curve) displays smaller values and a flatter profile in the region Lnk, pointing to a greater disorder than the other methods, but nevertheless showing some peaks of order.

The analysis of likelihood maps produced by TALOS-N allowed us to determine $\phi$, $\psi$ boxes which agree with the secondary structure predictions made by TALOS-N. Relative order predictions indicate regions with residual order in Lnk.

### TAiBP provides a complete enumeration of Lnk conformations

The TAiBP approach described in Methods (Section “Enumeration of Lnk conformations”), has been used for generating Lnk conformations. The cumulative numbers ($N_{iBPrun}$) of individual iBP runs for each fragment vary from 422 up to 1534, most of them being larger than 1000 (Table 1A). The distribution of the number of solutions for each individual iBP calculation was plotted (Fig. 1E) according to the analyzed fragment. An important feature of these distribution is that the number of solutions for individual iBP runs are all in the range $10^3$–$10^6$, corresponding to values smaller than the maximum of requested solutions ($10^9$) meaning that all trees were completely explored. Most of the fragments display numbers of solutions in the range $10^3$–$10^5$. But the fragment 253–262 (orange curve) displays somehow a narrower range ($10^3$–$10^4$) whereas the fragment 236–247 (magenta curve) displays a shifted range ($10^4$–$10^6$). The narrower range of fragment 253–262 may arise from smaller boxes used for residues 253, 255, 256, 259 and 260 (Table S1 and Fig. 1B).

**Table 1:** TAiBP calculation. A. Peptide fragments sampling with iBP. Lnk was divided in 7 fragments spanning the residues 220–231 (Pept1), 229–238 (Pept2), 236–247 (Pept3), 245–255 (Pept4), 253–262 (Pept5), 260–272 (Pept6) and 270-2-80 (Pept7). $N_{iBPrun}$: cumulative numbers of iBP runs for all sampled $\phi$, $\psi$ boxes on a given fragment. $N_{iBPconf}$: cumulative numbers of generated conformations in the iBP runs for all parsed restraints on a given fragment. $N_{clust}$: number of conformations after the clustering using self-organizing maps^32^. B. Assembling the 7 fragments Pept1 to Pept7 spanning Lnk.

A. iBP step	Lnk residue range	$N_{iBPrun}$	$\log _{10}(N_{iBPconf})$	$N_{clustiBP}$
Pept1	220–231	1534	7.8	477
Pept2	229–238	424	7.0	331
Pept3	236–247	1171	8.6	350
Pept4	245–255	768	7.4	182
Pept5	253–262	1534	7.2	303
Pept6	260–272	1024	7.8	361
Pept7	270–280	768	7.7	175

B. Assembly step	$N_{trials}$	$N_{clashes}$	$N_{saved}$	$N_{clust}$
Pept1-2	157,887	15,103	142,784	184
Pept1-2-3	64,400	7406	56,994	190
Pept1-2-3-4	34,580	3432	31,148	165
Pept1-2-3-4-5	49,995	4328	45,667	205
Pept1-2-3-4-5-6	74,005	12,615	61,390	234
Pept1-2-3-4-5-6-7	40,950	5953	34,997	215

Each assembly step is labeled “Pept” followed by the index of assembled fragments and describes the addition of the last index fragment to the protein obtained at the previous assembly step. $N_{trials}$: number of trials during the assembly run. $N_{clashes}$: number of solutions removed because of C$\alpha$ atoms closer than 1 Å. $N_{saved}$: number of solutions before clustering. $N_{clust}$: number of solutions after clustering using self-organizing maps^32^.

The cumulative numbers of iBP solutions ($\log _{10}(N_{iBPconf})$) are located (Table 1A) in the $10^{7.2}$–$10^{7.8}$ range, except the fragment Pept3 (residues 236-247) which displays a cumulative number of solutions ten times larger than other fragments, in agreement with the largest range of numbers of solutions observed for the corresponding individual iBP runs (magenta curve in Fig. 1E). A ratio of 2.7 is observed between the largest and smallest numbers of representative conformations ($N_{clustiBP}$) obtained after clustering by the self-organizing maps^32^, whereas this ratio was of about 4 for the initial numbers of iBP solutions $N_{iBPconf}$. This proves that the clustering has a key influence on the combinatorial of the iBP calculation.

The various steps of fragment assembly were analyzed (Table 1B) by monitoring several parameters: $N_{trials}$, $N_{clashes}$, $N_{saved}$ and $N_{clust}$. $N_{trials}$ is the number of assembly attempts equal to the product of the number of conformations available for each assembled fragment and displays quite different values depending on these numbers. Interestingly, the number of cases where atoms C$\alpha$ are closer than 1 Å ($N_{clashes}$) is for most cases about 10% of the number of assembly trials ($N_{trials}$), except for the assembly of the fragment Pept6 (residues 260–272), where the number of clashes is larger, about 15%. Interestingly, this fragment is the only one including two $\alpha$ helix predictions for R266 and R267. Discarding conformations for which C$\alpha$ are closer than 1 Å, which represents a very simple geometric condition is thus quite efficient to reduce the number of solutions. A ratio of 1.4 is observed between the largest and smallest numbers of representative conformations ($N_{clust}$) obtained from self-organizing maps, while this ratio was 4.6 for the numbers of trials $N_{trials}$. The clustering has even a greater influence on the combinatorial during the assembly step than during the iBP step.

After having finished the assembly, the last clustering step provides 215 conformations, which will be starting point to build the tandem domain protein and optimizing it along other independent measurements: residual dipolar couplings (RDC), paramagnetic resonance enhancements (PRE), small angle X-ray scattering (SAXS).

### Optimizing P1LnkP2 with respect to independent NMR parameters

Starting from the 215 Lnk conformations generated by TAiBP, the procedure to assemble P1 and P2 domains, described in Section “Assembling P1 and P2 to Lnk” of Methods, produced 83 conformations of P1LnkP2.

The fit to residual dipolar couplings (RDC) of the relative orientation of P1 and P2 domains within P1LnkP2 was evaluated by calculating theoretical RDCs using PALES 2.1^33^, as well as their correlation factors R with experimental RDCs. Correlation factors in the 0.59-0.82 range were obtained, and XPLOR-NIH^34^ was used to optimize with respect to RDCs the 33 conformations for which a correlation factor smaller than 0.7 was observed. The optimization along RDCs is described in the Section “Optimization of P1LnkP2 conformations along the RDCs” of Supplementary Materials. After optimization, the average correlation of the 33 conformations went up to 0.95 and all correlations were in the range 0.7- 0.98.

The conformations refined along RDCs were replaced among the 83 total conformations, and the 10 conformations (13, 20, 55, 145, 146, 150, 153, 160, 174, 186) displaying a distance between geometric centers of P1 and P2 smaller than 32 Å, were submitted to a second optimization round using XPLOR-NIH with respect to PREs as described in Supplementary Materials (Section “Refinement of P1LnkP2 conformations along the PREs”). This second refinement intended to improve the fit of PRE profiles by moving closer the domains P1 and P2.

**Figure 2:**
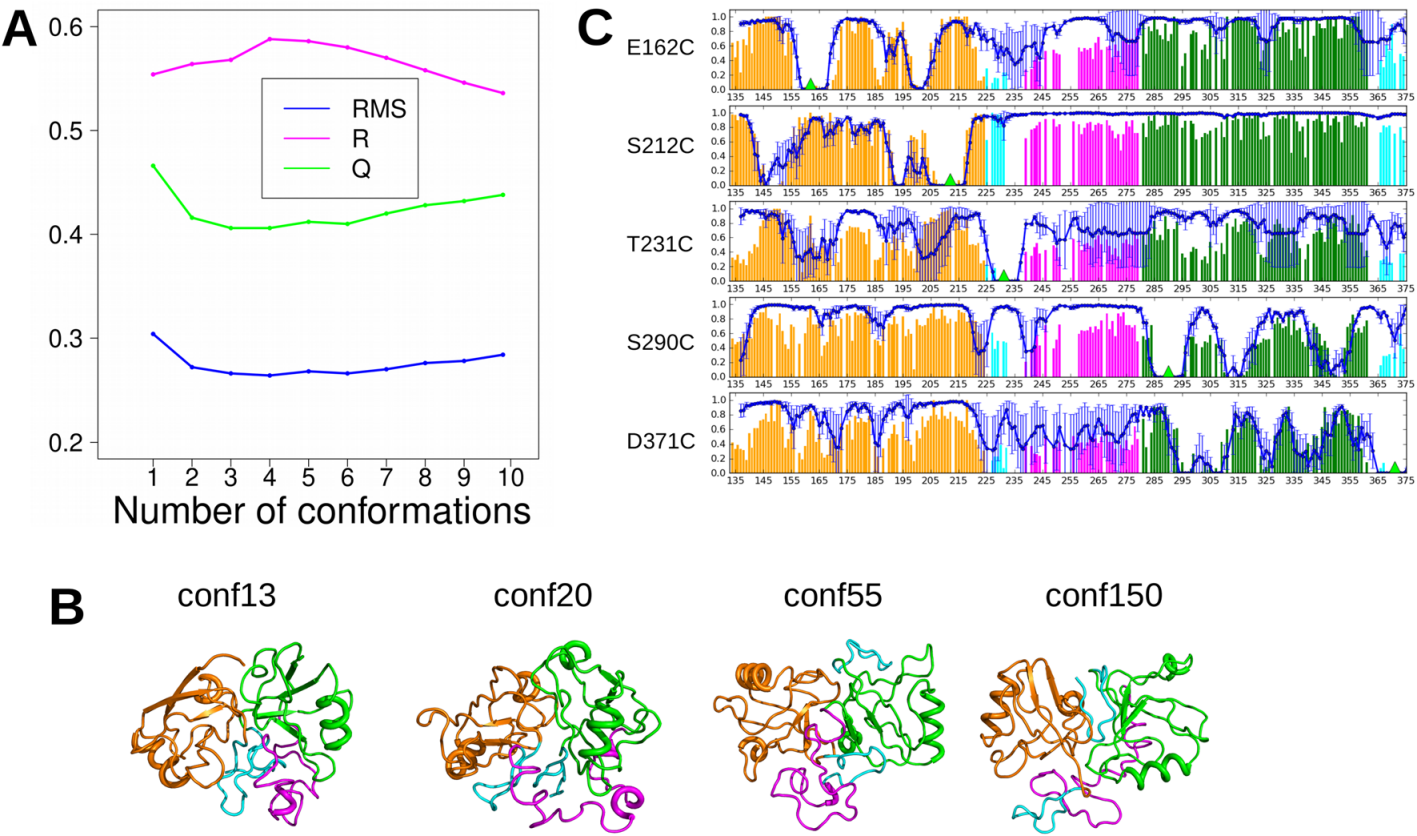
Selection of the P1LnkP2 conformations refined with respect to PRE data. A. Plot of the RMS, R and Q factors in function of the added conformation in the pool of selected closed conformations. The plot was prepared using R 3.4.1^58^. B. Conformations 13, 20, 55 and 150 detected in the group of best four conformations. They are drawn in cartoon with P1 colored in orange, P2 in green, Lnk in magenta and the two hairpins in cyan. The conformations were drawn using pymol 1.8.4.0^59^. C. Comparison of theoretical and experimental PRE profiles for conformations 13, 20 and 55. The theoretical profiles are plotted in blue with error bars and calculated assuming that the three conformations have the same populations. The experimental profiles are drawn as bars, colored with the same color-code than for the protein conformations. Each individual profile plot corresponds to one position of the MTSL probe from top to bottom: E162C, S212C, T231C, S290C, and D371C. The green triangles give the positions of the probe in the sequence. The plot was prepared using python script based on numpy 1.7.1^49^ and Matplotlib 1.5.3^60^ packages.

A manual inspection of the fits of PRE on each of the conformations refined along PRE revealed that no single conformation was able to provide a satisfying fit of all experimental PRE profiles (data not shown). Thus, the fit to PREs of all possible combinations of sub-groups of conformations has been analyzed. The set of P1LnkP2 conformations best suited to the PRE profile was built iteratively by choosing at each step to add the conformation such that the best correlation factor R was observed for the new set of conformations (Fig. 2A). In that way, a group of conformations was progressively built starting from conformation 13, then adding conformations 55, 20 and 150. RMS, R and Q values of 0.3, 0.6 and 0.4 were observed for this group. The plot of correlation factor R, of RMS and Q factors (Fig. 2A) along the size of conformation set shows that the minima of R, RMS and Q were observed for a group size of 4. Thus, the conformations 13, 20, 55 and 150 (Fig. 2B) were selected as closed conformations for further analysis.

### Determining the populations of conformations by fitting the SAXS curve

Theoretical SAXS curves were calculated on the P1LnkP2 conformation with the tool CRYSOL^35^ available in the package ATSAS 3.0.3^36^ using the procedure described in Section “Determination of the P1LnkP2 populations” of Supplementary Materials. The software BioEn 0.1.1^37^ and Mesmer 1.0.0^38^ were then used to determine the sets of populated conformations and their corresponding populations fitting the experimental SAXS curve. as described in the Section “Determination of the P1LnkP2 populations” in the Supplementary Materials. The set of processed conformations includes: (1) the $83-10=73$ P1LnkP2 conformations for which the distance between the geometric centers of P1 and P2 is larger than 32 Å; (2) various sets of conformations from the subset of closed conformations 13, 20, 55 and 150 previously selected after the PRE refinement step. Two series of BioEn calculations were performed using the whole SAXS curve (Table 2) or using the SAXS curve measured for scattering vectors *q* up to 3.5 $\hbox {nm}^{-1}$ (Table S7).

**Table 2:** Populations of conformations determined using BioEn 0.1.1^37^ on the 73 P1LnkP2 conformations for which the distance between the geometric centers of P1 and P2 was larger than 32 Å and various closed conformations among 13, 20, 55 and 150, previously selected according to the fit of PRE data (Fig. 2).

	conf13	conf20	conf55	conf13,20,55	conf13,20,55,150
conf12	$3.4 \pm 1.2$	–	$4.2 \pm 6.1\hbox {e}{-}3$	$4.2 \pm 3.4\hbox {e}{-}3$	$72.0 \pm 4.1\hbox {e}{-}4$
**conf13**	${\mathbf{9.9}} \pm {\mathbf{0.11}}$	**not incl**	**not incl**	**–**	**–**
**conf20**	**not incl**	${\mathbf{9.0}} \pm {\mathbf{0.16}}$	**not incl**	**–**	**–**
conf24	$15 \pm 0.38$	$18 \pm 1.2$	$16 \pm 2.9\hbox {e}{-}3$	$16 \pm 1.1\hbox {e}{-}03$	–
**conf55**	**not incl**	**not incl**	${\mathbf{10}} \pm {\mathbf{6.9e}}{-}{\mathbf{4}}$	${\mathbf{10}} \pm {\mathbf{4.0e}}{-}{\mathbf{4}}$	${\mathbf{17}} \pm {\mathbf{1.2e-}} \mathbf{4}$
conf140	$8.6 \pm 0.044$	$8.1 \pm 0.22$	$8.8 \pm 2.2\hbox {e}{-}4$	$8.8 \pm 3.8\hbox {e}{-}4$	$11.0 \pm 1.2\hbox {e}{-}4$
**conf150**	**not incl**	**not incl**	**not incl**	**not incl**	**–**
conf176	$63 \pm 0.94$	$65 \pm 1.3$	$60 \pm 4.6\hbox {e}{-}3$	$60 \pm 2.8\hbox {e}{-}3$	–
Final $\chi$2	$1.7 \pm 2.5\hbox {e}{-}7$	$2.7 \pm 7.1\hbox {e}{-}7$	$2.0 \pm 1.0\hbox {e}{-}6$	$3.2 \pm 1.6\hbox {e}{-}6$	$5.4 \pm 1.5\hbox {e}{-}6$
Final S	$-2.4\hbox {e}{-}6 \pm 2.1\hbox {e}{-}10$	$-1.3\hbox {e}{-}5 \pm 6.0\hbox {e}{-}10$	$-2.8\hbox {e}{-}5 \pm 8.9\hbox {e}{-}10$	$-7.7\hbox {e}{-}05 \pm 1.3\hbox {e}{-}9$	$-4.7\hbox {e}{-}5 \pm 1.3\hbox {e}{-}9$

After ten runs starting from random values of populations and performed on the whole set of conformations, all conformations for which the sum of populations over the ten runs was larger than 0.01 were gathered, and a second run of ten additional BioEn calculations was performed on this reduced set of conformations. The average and standard deviation values of populations obtained for each selected conformation from the second set of BioEn runs, are given in the Table, along with the final average values of $\chi ^2$ and of entropy *S*. In each calculation, the conformations tagged as “not incl” have not been initially included in the calculation, whereas the conformations tagged as “–” were included in the calculation, but not selected by BioEn^37^. The conformations belonging to the set of closed conformations are written in bold.

The first conclusion which can be drawn from the BioEn calculations (Tables 2 and S7) is that whatever is the set of included closed conformations, only one of them displays a non-null population except for the calculation conf13,20,55,150 in Table S7. This population is most of the time around 10%, and is equal to 17% (Table 2) or 13% (Table S7) for conformation 55 in the case when the four closed conformations 13, 20, 55 and 150 are included in the calculation. The SAXS fitting is thus no sensitive to the presence of a specific closed conformation and a population of about 10% globally observed for all closed conformations has to be interpreted as the global population of all closed conformations.

The calculation performed using the four closed conformations (last right column of the Tables 2 and S7) displays a larger final $\chi ^2$ value than other calculations and shows a large variation in the populations. Indeed, the conformation 176 which displays a population around 60% for all other calculations, is discarded from the final set of conformations. Similarly, the conformation 12, low populated in all other calculations, jumps up to 72%. Due to the inconsistency with other calculations observed when the conformation 150 is included, this conformation was discarded from the set of closed conformations.

In the first four columns of Tables 2 and S7, very similar population profiles are obtained, the biggest variations being observed between conformations 12 and 24. As the smallest average final value of $\chi ^2$ is observed for the calculations including only the conformation 13, the populations obtained in Table 2 for that case were kept as the result of the fitting of SAXS curve. The reduction of SAXS curve to the scattering vectors *q* up to $3.5\,\hbox {nm}^{-1}$ produce population results (Table S7) similar to those obtained with the full SAXS curve (Table 2). In addition, the superpositions of experimental and theoretical SAXS curves are quite similar in all Bioen calculations (Figure S2).

The software Mesmer^38^ was used in parallel to produce an independent estimation of populations. Two sets of Mesmer calculation were performed using different numbers of randomly chosen initial conformations (the so-called components) equal to 40 and 60. The same sets of conformations were selected by the two calculations, with very similar populations (Table S2). Interestingly, these conformations are also the ones selected by BioEn, except that the closed selected conformation is 150. The populations of conformations determined by Mesmer are also similar to the ones obtained by BioEn. One can notice a slight decrease of the population of 176 from about 60% to about 50%, and a change of population balance between conformations 12 and 24.

The fitting of SAXS curve using the software BioEn 0.1.1^37^ Mesmer 1.0.0^38^ and ATSAS 3.0.3^36^ select the same set of conformations and produce similar results for the populations of conformation. Nevertheless, the SAXS curve fitting is more reliable for small than for large scattering vectors. Unsurprisingly, it was not possible to determine the relative population of each closed conformations, as all closed conformations display similar global shapes.

### The conformational landscape of P1LnkP2

**Figure 3:**
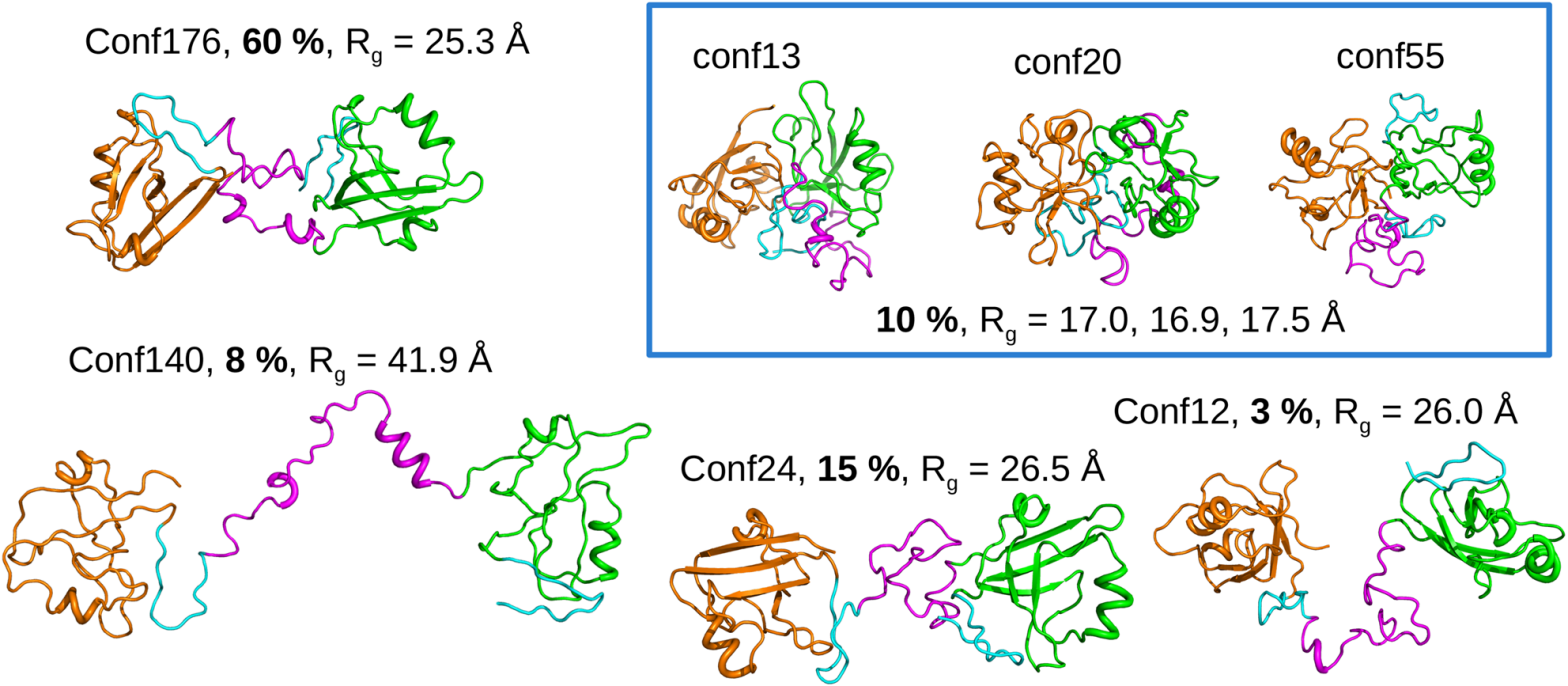
Overview of the P1LnkP2 conformations selected by BioEn 0.1.1^37^. The conformation indexes are given along with the populations obtained by SAXS curve fitting in bold, and with the gyration radius **REV2NUMB6** ($\hbox {R}_g$). The most closed P1LnkP2 conformations, for which it was not possible to determine the individual populations from the fitting of SAXS data, are plotted within a frame. The conformations were drawn using pymol 1.8.4.0^59^.

The previous analyses allow us to propose the following set of conformations for describing the conformational landscape of P1LnkP2 in solution (Fig. 3). The most populated conformation 176, with semi-closed configuration of P1 and P2 displays a population of 60% or 50% depending on the algorithm used for population determination. Closed conformations display globally a population of 10%, but the SAXS fitting does not allow to determine the relative proportion of each closed conformation within this global value. Other conformations, 24 and 12, displays an open P1-P2 configuration with respective populations of 15 and 3% or 7 and 20% depending on the use of BioEn or Mesmer. The very extended conformation 140 displays a population around 10% whatever procedure used to determine the populations.

Using the individual gyration radii of the individual conformations along with their relative populations (Fig. 3), an average value of gyration value can be estimated to be 26 Å, in total agreement with the gyration radius of 27.5 Å previously^27^ measured. The small discrepancy of 1.5Å between these two values could be assigned to solvation layer present in solution around the protein and not taken into account in the conformations determined in the present work.

The fit of PRE data by the three selected closed conformations 13, 20 and 55 can be evaluated by the comparison of experimental and theoretical PRE profiles (Fig. 2C) realized using the same relative populations for the three conformations. The theoretical profiles displays variations in reasonable agreement with the experimental profiles. RMS, R and Q factors have been calculated for each MSTL spin label and each closed conformation (Table S3). For most spin labels and protein conformations, reasonable R correlations were obtained.

**Figure 4:**
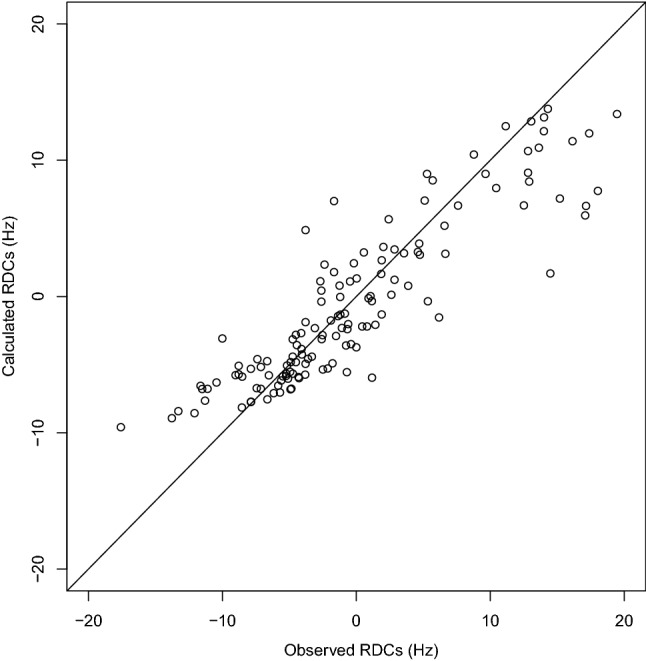
Comparison of the calculated and experimental residual dipolar coupling (RDC) values. The RDC values have been calculated with the software PALES 2.1^33^ on the conformations 12, 13, 24, 140, 176 and were averaged using the populations determined in Fig. 3. The plot was prepared using R 3.4.1^58^.

The RDC values have been recalculated on the conformations 12, 13, 24, 140, 176 using the software PALES and averaged according to populations displayed in Fig. 3. The comparison of calculated and observed RDC values (Fig. 4) reveals a reasonable agreement between the two sets of values, with a RMS of 4.1 Hz, a R factor of 0.8 and a Q factor of 0.5. The analysis of the agreement with RDCs for individual conformations (Table S4) reveals R correlation factors larger than 0.9 for all closed conformations and conformation 140 and larger than 0.8 for conformation 176.

### Consistency between the observed and theoretical likelihood maps

The consistency of the obtained set of conformations and populations was probed with respect to the likelihood ($\phi$, $\psi$) maps $\hbox {M}_{{obs}}$ predicted by TALOS-N from experimental chemical shifts and initially used to get the $\phi$ and $\psi$ boxes (Fig. 1B). Theoretical chemical shifts have been calculated from the conformations 12, 13, 24, 140 and 176 for nuclei H$\alpha$, HN, $^{15}$N, $^{13}$C$\alpha$, $^{13}$C$\beta$ (when present in the residue) and $^{13}$C’ using the software Shiftx 1.0^39^. The chemical shifts were averaged according to the population of individual conformations (Fig. 3), assuming fast equilibrium between the conformations. The obtained averaged chemical shifts were then analyzed using TALOS-N and the obtained likelihood maps $\hbox {M}_{{theo}}$ compared to the initial maps $\hbox {M}_{{obs}}$.

The contour plots of maps $\hbox {M}_{{theo}}$ and $\hbox {M}_{{obs}}$ are superimposed for each Lnk residue using black color for $\hbox {M}_{{obs}}$ and red color for $\hbox {M}_{{theo}}$ (Figure S3). Inspection of the plots reveals a good agreement between $\hbox {M}_{{obs}}$ and $\hbox {M}_{{theo}}$ maps. Indeed, over the 61 residues considered, only 17 display discrepancies for the peak positions in the two maps. Two glycines, G228 and G256, display a displacement of the peak located in the region of positive $\phi$ values. As glycine residues display a tendency to populate regions of positive $\phi$ values, this may bias the TALOS-N database and consequently the predicted likelihood map. In addition, several residues display peak variations in the $\alpha$ and $\beta$ regions of the maps. The residues Y235, T236, Q253 and E278 display on maps $\hbox {M}_{{obs}}$ (black) two peaks in the regions of $\alpha$ helix and $\beta$ strand, and the $\alpha$ peak disappears in maps $\hbox {M}_{{theo}}$ (red). At the contrary, the residues S249, Q253, H255, as well as the residues 260-264 and 269-272, display also two peaks on maps $\hbox {M}_{{obs}}$ (black) in the regions of $\alpha$ helix and $\beta$ strand, and the $\beta$ peak disappears in maps $\hbox {M}_{{theo}}$ (red). One should notice that only one or two chemical shifts are assigned in H233, V238 and H255, and that the of residues W237, P247, and 252-254 are unassigned: the lack of measured chemical shifts makes the map $\hbox {M}_{{obs}}$ to rely mainly on sequence alignment with the database of TALOS-N and could hamper the precision of $\hbox {M}_{{obs}}$ prediction. We should also notice that for the residues displaying peak variations in regions $\alpha$ and $\beta$ of maps $\hbox {M}_{{theo}}$ and $\hbox {M}_{{obs}}$, the 83 P1LnkP2 conformations displays backbone dihedral angles in the two regions. But, after determining populations by fitting of SAXS curve, the distribution of $\psi$ angle values is shifted toward one of the $\alpha$ or $\beta$ regions. Most of the discrepancies between $\hbox {M}_{{theo}}$ and $\hbox {M}_{{obs}}$ peaks in $\alpha$ and $\beta$ Ramachandran regions is thus a consequence of the fitting of SAXS data.

## Discussion

In the present work, a protocol for systematically enumerating the conformations of a tandem domain protein has been presented. This protocol makes use of the TAiBP approach^19,20^ which is specifically designed for partially disordered protein regions. In TAiBP, a reformulation of the distance geometry approach permits an exhaustive sampling of protein conformations within a given set of distance and angle limits. The availability of all possible conformations of Lnk consequently provides the largest possible exploration of the relative positions of P1 and P2 in the space. The subsequent refinements of the P1LnkP2 conformations with respect to RDC and PRE measurements along with the population determination by fitting of SAXS curves produces a exhaustive set of conformations verifying all experimental measurements. The reliability of this set of conformations arises directly from the exhaustive exploration allowed by TAiBP. In this frame, one should point out that the convergence of calculation is not expected to validate the set of obtained conformations, as the approach proposed here for conformational space exploration is not based on a local optimization methods. The procedure proposed here requires at least the knowledge of heteronuclear NMR assignment and chemical shifts as well as the measurement of SAXS data.

The backbone angle limits used as inputs for Lnk conformational analysis are determined using a new point of view on the processing of likelihood maps outputs of the neural network TALOS-N^28^. These maps are transformed into probability maps and boxes of $\phi$ and $\psi$ values are extracted from all probability regions larger than a given threshold. These boxes will be then used to sample Ramachandran multiple regions. As the bounds for TAiBP calculations are exclusively determined by geometric parameters, the Lnk conformations do not take into account the free energy aspects. The fitting of SAXS curve then permits to obtain the relative populations of the P1LnkP2 conformations, re-introducing the free energy information.

At the end of calculation, a limited number of discrepancies between observed and theoretical likelihood ($\phi$, $\psi$) maps, concerns only 17 residues over 61. The discrepancies could arise from several reasons. An obvious one is that TALOS-N was designed on systems displaying a unique basin of conformations. Another one was pointed out above: the fitting of SAXS curve induce the selection of conformations from specific Ramachandran regions and their corresponding maps $\hbox {M}_{{theo}}$ are different from the $\hbox {M}_{{obs}}$ maps. Nevertheless, the small quantity of residues displaying discrepancies is of good hope for the quantitative analysis of TALOS-N likelihood maps. Beside, it should be noticed that cross-correlated spin relaxation was recently proposed^40,41^ for directly determining probability distributions of $\phi$ and $\psi$ angles: these distributions could be also used for determining $\phi$ and $\psi$ boxes for enumeration of conformations. In addition, backbone angle information determined from J couplings could be used as inputs for the TAiBP calculation.

The refinement of P1LnkP2 conformations with respect to the NMR and SAXS measurements put in evidence the different influence of global (SAXS) and more local (RDC, PRE) parameters. In particular, the fitting from SAXS data is much less sensitive to closed conformations than the fitting to PRE data. Discrepancy of SAXS measurements with other biophysical techniques is a well-known aspect of studies on partially disordered proteins^42,43^.

The obtained conformations and population gives a simplified representation of the conformational landscape of P1LnkP2. This landscape is in qualitative agreement with the conformational distribution determined in the previous structural study of the whirlin tandem domain^27^, the mostly populated conformation being the one with P1 and P2 domain close to each other. The most closed conformations, in agreement with the PRE measurements are less populated than semi-closed ones. This may be related to a conformational entropy barrier. Indeed, as the Lnk region is more mobile than the P1 and P2 domains, Lnk contributes importantly to the conformational entropy. The Lnk packing requires a decreasing of conformational entropy inducing consequently an entropy barrier, which could explain the lowest populations of closed conformations.

## Methods

The P1LnkP2 conformations have been determined in two steps: first using the TAiBP approach for systematically enumerating Lnk conformations, then applying a specifically designed procedure to assemble P1 and P2 to the Lnk conformations. The scripts and programs required to run TAiBP are available at: https://github.com/tmalliavin/.

### Enumeration of Lnk conformations

The Lnk conformations have been enumerated using the threading-augmented interval branch-and-prune (TAiBP) approach recently proposed^20^ to take advantage of the systematic enumeration permitted by the iBP approach^11–15^ while overcoming the combinatorial barrier arising from the enormous space of protein conformations^44^. The approach efficiency was proved for proteins up to 60 residues^20^ and TAiBP is thus relevant for the Lnk calculation, which contains 61 residues. TAiBP is composed two steps: (1) individual iBP calculations of peptide fragments spanning the studied protein; (2) enumeration of Lnk conformations by systematic assembly of fragment conformations.

The enumeration of Lnk conformations was performed using boxes of backbone angles $\phi$ and $\psi$. These boxes (Fig. 1B and Table S1) have been extracted from the likelihood Ramachandran maps obtained by TALOS-N^28^ as described in Results. The enumeration of conformations in the iBP approach is based on the building of a tree, each node of the tree corresponding to a position of an atom. The tree building allows one to enumerate the various possibilities for atom positions (branching step) whereas additional geometric information is used to accept or reject each newly built branch (pruning step). The atoms are positioned from the position of previous atoms according to a specific ordering (Table S5) of the protein atoms^13^ and using the iBP algorithm described by Worley et al^15,19^. The atom ordering ensures that there is a restricted and manageable locus for the spatial position of every atom. This is achieved using a “relaxed form” of trilateration with respect to the three preceding atoms in the order. More precisely, two out of three of the distances involved in trilateration must be known exactly, and one may be subject to uncertainty and represented by an interval^13,15^. As described in Section “Calculation of the backbone angles from interatomic distances” in the Supplementary Material, the backbone dihedral angles $\phi$ and $\psi$ can be straightforwardly related to bond lengths and bond angles and respectively to distances between atoms C of residues $i-1$ and *i* and between atoms N of residues *i* and $i+1$. This equivalence between the backbone dihedral angles and interatomic distances permits to use the angles $\phi$ and $\psi$ for the so-called branching step. Once atoms positions have been generated in the tree, each one is examined to check whether they verify pruning geometric information and is pruned in the case the information is not verified. In the present calculation, the pruning was applied by preventing atoms to be closer than the sum of their van der Waals radii and by checking that the improper angle values are correct. The bond lengths, bond angles, improper angles and van der Waals radii were taken from the force field protein-allhdg5-4 PARALLHDG (version 5.3)^45^ (Table S6). The van der Waals radii were scaled by a factor of 0.7.

The $\phi$/$\psi$ boxes determined for each Lnk residue from TALOS output were systematically combined by permutation to prepare individual iBP calculations, realized in a way similar to those described in Ref.^20^. Lnk was divided in 7 fragments corresponding to residues 220-231, 229-238, 236-247, 245-255, 253-262, 260-272 and 270-280. For each fragment, two dummy residues were added at the N and C terminal extremities, and the $\phi$ and $\psi$ dihedral angles of the inner peptide residues were sampled according to the box limits (Table S1). In order to avoid pruning due to slight discrepancy between distances, a tolerance of 0.05 Å has been added to the bounds of distance intervals. The maximum number of branches by interval was set to 4. The minimum discretization factor, which is the minimum ratio between each distance interval to the number of tree branches generated within the interval, was set to 0.1 Å, in order that the branching does not over-sample small intervals. The iBP algorithm was used to exhaustively enumerate the conformations verifying these limits by systematically exploring the tree defined by the boxes on $\phi$ and $\psi$ angles. The number of saved conformations was reduced by enforcing that two successively saved conformations display a coordinate RMSD larger than 2 Å. A maximum number of $10^9$ saved conformations was permitted for each iBP run. The solutions were stored in a multiframe dcd format^46^.

The generated conformations of neighbouring peptide fragments in the protein sequence were then assembled by superimposing the three last and initial residues of the fragments successive in the sequence. The dummy N and C terminal residues added at the step of individual iBP calculations were not included in the superimposition and are removed after the assembly step. During the superimposition of backbone atoms located in the three superimposed residues, the atom number for which the smallest distance was observed between corresponding atoms in the two peptides was used to decide where to stop with the first peptide and to continue with the second one. The assembled conformation was then checked to verify whether there is no C$\alpha$ atoms closer than 1 Å. The assembled conformations not verifying this condition were pruned from the calculation. The fragment assembly was implemented using python scripting based on the MDAnalysis 0.1^47,48^ and numpy 1.7.1^49^ packages.

To scale down the combinatorial explosion of the calculation, a clustering approach, the **S**elf-**O**rganizing **M**ap (SOM)^32,50–52^, which is an artificial neural network (ANN) trained using unsupervised learning, was used to reduce the number of conformations. SOM displays the advantage with respect to the k-means clustering approach that it does not require the predetermined knowledge of the number of clusters. The SOM approach was used after each iBP calculation or assembly step as soon as the number of saved conformations was larger than 1000. The conformations are encoded from the distances $d_{ij}$ calculated between the *n* $\hbox {C}_\alpha$ atoms by diagonalizing the covariance matrix *C*:

1 $$\begin{aligned} C_{i,j} =\frac{1}{n}\sum _{k=1}^{n}\sum _{l=1}^{n} (d_{i,k}-\bar{d_i})(d_{l,j}-\bar{d_j}) \end{aligned}$$

where $\bar{d_s} = \frac{1}{n}\sum _{p=1}^{n}d_{s,p}$. The information contained in the matrix *C* is equivalent to its four largest eigenvalues along with the corresponding eigenvectors, corresponding to an input vector of length 4($n+1$). The input vectors obtained on the saved conformations are used to train a periodic Euclidean 2D self-organizing map (SOM), which corresponds to a three-dimensional matrix. The first two matrix dimensions were chosen to be $100\times 100$ and define the map size, the third dimension being equal to 4($n+1$). Each vector along the third dimension defines a neuron of the map. The neurons of the self-organizing map are initialized with a random uniform distribution covering the range of values of the input vectors previously obtained from the covariance matrix *C*. At each step, an input vector is presented to the map, and the neuron closest to this input is updated. The maps were trained as previously described^32,53^.

Once the SOM has been determined, representative conformations are extracted from the conventional **U**nified **d**istance **m**atrix (U-matrix) calculated from the final SOM neurons. For each neuron $\nu$, the corresponding U-matrix element is calculated as the average Euclidean distance between the neuron $\mu$ and its eight immediate neighbors:

2 $$\begin{aligned} \text{ U-matrix }(\mu ) = \frac{1}{8}\sum _{\nu \in N(\mu  )}d({\nu },{\mu }) \end{aligned}$$

where $N(\mu )$ is the set of neighbors, and $d(\nu ,\mu )$ is the Euclidean distance between the neurons $\mu$ and $\nu$. The neurons corresponding to local minima of the U-matrix, and thus to local maxima of conformational homogeneity, are extracted and the protein conformation being first stored in that neuron is saved. The conformations generated during the iBP or assembly steps are finally replaced by the sets of representative conformations extracted from local minima of U-matrix.

### Assembling P1 and P2 to Lnk

The PDZ domains P1 and P2 were added to each Lnk conformation according to the following procedure. The domain P1 extracted from the P1P2 HADDOCK complex^27^, was assembled to Lnk conformation (Figure S1A) by superimposing the backbone atoms of the N-terminal segment 232-238 of Lnk to the backbone atoms of the segment 232–238 present at the C terminal extremity of the domain P1 (Figure S1A). The superimposition was realized using a 3D grid generated within the following limits: the maximum and minimum values extracted from the atomic coordinates of atoms C$\alpha$ of Lnk, from which the gyration radius $R_g$ of the domain P1 was added and subtracted (Figure S1B). The grid points which were closer than $R_g$ from the atom C$\alpha$ of residue 238 in Lnk and which were more apart than 2 Å from any atom C$\alpha$ of Lnk, were selected for further processing.

Then, the geometric center of P1 was placed at each of the grid points previously generated. The two vectors $\hbox {V}_{{P1}}$ and $\hbox {V}_{{Lnk}}$ connecting the atoms C$\alpha$ of residues 232 and 238 in P1 and in Lnk were calculated and the 3D rotation matrix allowing to transform $\hbox {V}_{{P1}}$ into $\hbox {V}_{{Lnk}}$ determined (Figure S1C). This rotation matrix was then applied on the atomic coordinates of P1. The vectors $\hbox {V}_{{232}}$ (respectively $\hbox {V}_{{238}}$) connecting atom C$\alpha$ of residue 232 (respectively 238) in Lnk and P1 were calculated and the P1 atomic coordinates were translated along the vector: $\mathbf{T} = \frac{1}{2}(\mathbf{V}_{\mathbf{232}}+\mathbf{V}_{\mathbf{238}})$ (Figure S1D). The translated position of P1 was accepted according to two conditions: (1) the number of atomic clashes between the P1 and Lnk, determined as the number of C$\alpha$ pairs closer than 1 Å, was smaller than 3; (2) the average distances between atoms C$\alpha$ of residues 232 in Lnk and P1 and between atoms C$\alpha$ of residues 238 in Lnk and P1 were smaller than 6 Å. The P1-Lnk protein was then generated by merging the atomic coordinates of P1 for residues 136–238 and the atomic coordinates of Lnk for residue 239–280. Sidechains were generated for the linker in the selected solutions and molecular dynamics trajectories of 100 ps were recorded using the protocol described in Supplementary Material (Section “Molecular dynamics refinement in implicit solvent”) with positional restraints on the backbone heavy atoms for residues 225–280. At the end, conformations for which the total energy was higher than − 2000 kcal/mol were discarded, which produced 153 conformations of P1-Lnk.

The domain P2 was then assembled to P1Lnk. During the previous structural work on P1LnkP2^27^, a model of the complex P1/P2, in which the two PDZ domains were not covalently bound, was built using the HADDOCK server^54–56^ from the RDC restraints and the previously determined structures of the human whirlin PDZ domains (PDB entries 1UEZ and 1UF1). This model was used here to impose a starting point for the relative orientations of the P1 and P2 domains inside P1LnkP2. The P1P2 HADDOCK complex and the P1-Lnk (Figure S1E) conformations were superimposed along the backbone atoms of the residues 136–238, corresponding to P1 and the hairpin Hp1 (Figures 1A and S1F). Second, the vector $\hbox {V}_{{276}}$ relating the positions of the atom C$\alpha$ of residue 276 in the HADDOCK P1P2 complex and in the P1-Lnk conformation was calculated. A translation defined by this vector was applied to the domain P2 present in the complex P1P2 and the translated P2 was concatenated to P1-Lnk (Figure S1G). The number of clashes was determined as the number of atoms closer than 1 Å between residues 136-275 (P1-Lnk) and 276–376 (P2). If less than 50 clashes were observed, the construction was kept and submitted to a 100ps molecular dynamics, following the procedure described in Supplementary Material (Section “Molecular dynamics refinement in implicit solvent”). During that run, positional restraints on all atoms except in residues 274–278 were applied. After this step, 87 conformations of P1LnkP2 for which the total energy was better than − 2000 kcal/mol were selected, and after manual inspection, four additional conformations displaying knots were removed from the set of solutions. Finally, 83 conformations were kept to perform further refinement.

## Supplementary information

Supplementary material 1 (pdf 517 KB)

## References

[1] Dunker, A. K. *et al.* What’s in a name? Why these proteins are intrinsically disordered. *Intrinsically Disord Proteins* **1**, e24157 (2013). 10.4161/idp.24157 PMC5424803 28516007

[2] Csizmok V, Follis AV, Kriwacki RW, Forman-Kay JD (2016). Dynamic protein interaction networks and new structural paradigms in signaling. Chem. Rev..

[3] Bah A, Forman-Kay JD (2016). Modulation of intrinsically disordered protein function by post-translational modifications. J. Biol. Chem..

[4] Ali M, Simonetti L, Ivarsson Y (2020). Screening intrinsically disordered regions for short linear binding motifs. Methods Mol. Biol..

[5] Meyer K, Selbach M (2020). Peptide-based interaction proteomics. Mol. Cell. Proteomics.

[6] Yan J, Dunker AK, Uversky VN, Kurgan L (2016). Molecular recognition features (MoRFs) in three domains of life. Mol. BioSyst..

[7] Cumberworth A, Lamour G, Babu MM, Gsponer J (2013). Promiscuity as a functional trait: intrinsically disordered regions as central players of interactomes. Biochem. J..

[8] Feldman HJ, Hogue CW (2000). A fast method to sample real protein conformational space. Proteins.

[9] Bernadó P (2005). A structural model for unfolded proteins from residual dipolar couplings and small-angle X-ray scattering. Proc. Natl. Acad. Sci. USA.

[10] Jha AK, Colubri A, Freed KF, Sosnick TR (2005). Statistical coil model of the unfolded state: resolving the reconciliation problem. Proc. Natl. Acad. Sci. USA.

[11] Lavor C, Liberti L, Maculan N, Mucherino A (2012). The Discretizable Molecular Distance Geometry Problem. Comput. Optim. Appl..

[12] Lavor C, Liberti L, Mucherino A (2013). The interval Branch-and-Prune algorithm for the discretizable molecular distance geometry problem with inexact distances. J. Glob. Optim..

[13] Liberti L, Lavor C, Mucherino A, Mucherino L, Lavor C, Liberti L, Maculan N (2014). The discretizable molecular distance geometry problem seems easier on proteins. Distance Geometry: Theory, Methods and Applications.

[14] Liberti L, Lavor C, Maculan N, Mucherino A (2014). Euclidean distance geometry and applications. SIAM Rev..

[15] Lavor C, Alves R, Figueiredo W, Petraglia A, Maculan N (2015). Clifford algebra and the discretizable molecular distance geometry problem. Adv. Appl. Clifford Algebras.

[16] Cassioli A (2015). An algorithm to enumerate all possible protein conformations verifying a set of distance constraints. BMC Bioinf..

[17] Mareuil F, Malliavin TE, Nilges M, Bardiaux B (2015). Improved reliability, accuracy and quality in automated NMR structure calculation with ARIA. J. Biomol. NMR.

[18] Feldman HJ, Hogue CW (2002). Probabilistic sampling of protein conformations: new hope for brute force?. Proteins.

[19] Worley B (2018). Tuning interval branch-and-prune for protein structure determination. J. Global Optim..

[20] Malliavin TE, Mucherino A, Lavor C, Liberti L (2019). Systematic exploration of protein conformational space using a distance geometry approach. J. Chem. Inf. Model..

[21] Matsushima N (2008). Flexible structures and ligand interactions of tandem repeats consisting of proline, glycine, asparagine, serine, and/or threonine rich oligopeptides in proteins. Curr. Protein Pept. Sci..

[22] Troncoso, M. F., Elola, M. T., Croci, D. O. & Rabinovich, G. A. Integrating structure and function of ‘tandem-repeat’ galectins. *Front. Biosci. (Schol Ed)* **4**, 864–887 (2012). 10.2741/s305 22202096

[23] Sang M (2014). LIM-domain-only proteins: multifunctional nuclear transcription coregulators that interacts with diverse proteins. Mol. Biol. Rep..

[24] Shaw N, Liu ZJ (2014). Role of the HIN domain in regulation of innate immune responses. Mol. Cell. Biol..

[25] Troncoso MF (2014). Galectin-8: a matricellular lectin with key roles in angiogenesis. Glycobiology.

[26] Wang L (2019). Identification of whirlin domains interacting with ESPIN: A study of the mechanism of Usher syndrome type II. Mol. Med. Rep..

[27] Delhommel F (2017). Structural characterization of whirlin reveals an unexpected and dynamic supramodule conformation of its PDZ tandem. Structure.

[28] Shen Y, Bax A (2015). Protein structural information derived from NMR chemical shift with the neural network program TALOS-N. Methods Mol. Biol..

[29] Nielsen, J. T. & Mulder, F. A. There is diversity in disorder—“In all chaos there is a cosmos, in all disorder a secret order”. *Front Mol Biosci* **3**, 4 (2016). 10.3389/fmolb.2016.00004 PMC4749933 26904549

[30] Berjanskii MV, Wishart DS (2005). A simple method to predict protein flexibility using secondary chemical shifts. J. Am. Chem. Soc..

[31] Camilloni C, De Simone A, Vranken WF, Vendruscolo M (2012). Determination of secondary structure populations in disordered states of proteins using nuclear magnetic resonance chemical shifts. Biochemistry.

[32] Bouvier G (2014). Functional motions modulating VanA ligand binding unraveled by self-organizing maps. J. Chem. Inf. Model..

[33] Zweckstetter M, Bax A (2000). Prediction of sterically induced alignment in a dilute liquid crystalline phase: aid to protein structure determination by NMR. J. Am. Chem. Soc..

[34] Schwieters CD, Bermejo GA, Clore GM (2018). Xplor-NIH for molecular structure determination from NMR and other data sources. Protein Sci..

[35] Svergun DI, Barberato C, Koch M (1995). CRYSOL—a Program to evaluate X-ray solution scattering of biological macromolecules from atomic coordinates. J. Appl. Cryst..

[36] Manalastas-Cantos K (2021). ATSAS 3.0: expanded functionality and new tools for small-angle scattering data analysis. J. Appl. Crystallogr..

[37] Köfinger J (2019). Efficient ensemble refinement by reweighting. J. Chem. Theory Comput..

[38] Ihms EC, Foster MP (2015). MESMER: minimal ensemble solutions to multiple experimental restraints. Bioinformatics.

[39] Neal S, Nip AM, Zhang H, Wishart DS (2003). Rapid and accurate calculation of protein 1H, 13C and 15N chemical shifts. J. Biomol. NMR.

[40] Kauffmann C, Kazimierczuk K, Schwarz TC, Konrat R, Zawadzka-Kazimierczuk A (2020). A novel high-dimensional NMR experiment for resolving protein backbone dihedral angle ambiguities. J. Biomol. NMR.

[41] Kauffmann C, Zawadzka-Kazimierczuk A, Kontaxis G, Konrat R (2021). Using cross-correlated spin relaxation to characterize backbone dihedral angle distributions of flexible protein segments. ChemPhysChem.

[42] Borgia A (2016). Consistent view of polypeptide chain expansion in chemical denaturants from multiple experimental methods. J. Am. Chem. Soc..

[43] Gomes GW (2020). Conformational ensembles of an intrinsically disordered protein consistent with NMR, SAXS, and single-molecule FRET. J. Am. Chem. Soc..

[44] Levinthal C (1968). Are there pathways for protein folding?. J. Chem. Phys..

[45] Engh R, Huber R (1991). Accurate bond and angle parameters for X-ray protein structure refinement. Acta Crystallogr. A.

[46] Humphrey W, Dalke A, Schulten K (1996). VMD - Visual Molecular Dynamics. J. Mol. Graph..

[47] Michaud-Agrawal N, Denning EJ, Woolf TB, Beckstein O (2011). MDAnalysis: a toolkit for the analysis of molecular dynamics simulations. J. Comput. Chem..

[48] Gowers, R.J., *et al.* MDAnalysis: A Python Package for the Rapid Analysis of Molecular Dynamics Simulations. In S. Benthall and S. Rostrup, editors, *Proceedings of the 15th Python in Science Conference*, 98–105 (2016).

[49] Harris CR (2020). Array programming with NumPy. Nature.

[50] Kohonen T (1982). Self-organized formation of topologically correct feature maps. Biol. Cybern..

[51] Kohonen T (2001). Self-organizing Maps.

[52] Miri L (2014). Stabilization of the integrase-DNA complex by Mg2+ ions and prediction of key residues for binding HIV-1 integrase inhibitors. Proteins.

[53] Spill Y, Bouvier G, Nilges M (2013). A convective replica-exchange method for sampling new energy basins. J. Comput. Chem..

[54] Dominguez C, Boelens R, Bonvin AM (2003). HADDOCK: a protein-protein docking approach based on biochemical or biophysical information. J. Am. Chem. Soc..

[55] van Zundert GCP (2016). The HADDOCK2.2 web server: user-friendly integrative modeling of biomolecular complexes. J. Mol. Biol..

[56] Saponaro A, Maione V, Bonvin AMJJ, Cantini F (2020). Understanding docking complexes of macromolecules using HADDOCK: the synergy between experimental data and computations. Bio Protoc.

[57] Foundation TD. LibreOffice [Internet]. 2020. Available from: https://www.libreoffice.org/.

[58] R Core Team. *R: A Language and Environment for Statistical Computing*. R Foundation for Statistical Computing, Vienna, Austria (2017).

[59] Warren L. DeLano. *The PyMOL Molecular Graphics System, Version 1.2r3pre*. Schrödinger, LLC.

[60] Hunter JD (2007). Matplotlib: a 2D graphics environment. Comput. Sci. Eng..

