# Supplementary material for: Tandem domain structure determination based on a systematic enumeration of conformations
Source: Sci Rep. 2021 Aug 19;11:16925. doi: 10.1038/s41598-021-96370-z (PMC8376923; doi:10.1038/s41598-021-96370-z)
Supplement: Supplementary file 1 — Supplementary material 1 (pdf 517 KB) [file 41598_2021_96370_MOESM1_ESM.pdf]

Supplementary material: Tandem domain structure determination based on a systematic enumeration of conformations

Thérèse E. Malliavin*

Unité de Bioinformatique Structurale, Institut Pasteur, UMR 3528, CNRS, Departement de Bioinformatique, Biostatistique et Biologie Intégrative, Institut Pasteur, USR 3756, CNRS, Paris, (75015) France

E-mail: therese.malliavin@pasteur.fr

1 Experimental measurements

All experimental data have been measured and described in the previous study of tandem domain protein by Delhommel et al.[1] The experimental parameters used here for the calculation of P1LnkP2 conformations are: (i) the nuclear magnetic resonance (NMR) chemical shifts of the nuclei ^{1}H, ^{15}N, ^{13}C of Lnk residues; (ii) the H^{N}-N residual dipolar couplings (RDCs) obtained on the P1 and P2 domains, from the Pf1-phages alignment medium; (iii) the paramagnetic relaxation enhancements (PRE) obtained by conjugation of an MTSL probe in five individually mutated positions (E162C, S212C, T231C, S290C, and D371C); (iv) the small-angle X-ray scattering (SAXS) curve measured on P1LnkP2.

The small-angle X-ray scattering (SAXS) data were collected at the BioSAXS beamline BM29 at the European Synchrotron Radiation Facility (Grenoble, France) and at the SWING beamline at Soleil (Saclay, France). The sample was concentrated from 8.8 to 11.2 g/l prior

to the experiments, and scattering vectors q were ranging from 0.119 to 4.007 nm^{-1}. More information is given in the section "Method details" in Delhommel et al.[1]

2 Molecular dynamics refinement in implicit solvent

Molecular dynamics (MD) trajectories were used to relax several systems during the procedure of assembling domains P1 and P2 to Lnk. The MD trajectories were recorded using NAMD 2.13.[2] Topology parameters were taken from the force fields c36[3] and c36m.[4] The simulations were performed in Generalized Born implicit solvent (GBIS)[5] at a temperature of 300 K. A ion concentration of 0.3M, and a cutoff of 12 Å for calculating Born radius were used. A cutoff of 14 Å and a switching distance of 13 Å were defined for non-bonded interactions. The RATTLE algorithm[6,7] was used to keep all covalent bonds involving hydrogens rigid, enabling a time step of 2 fs. Temperature was regulated according to a Langevin thermostat.[8] At the beginning of each trajectory, the system was first minimized for 1,000 steps, then heated up gradually from 0 K to 300 K in 30,000 integration steps. Finally, the system was equilibrated for 5,000 steps. During all steps, from minimization to production, positional restraints were applied on specific regions of the system with a constant force of 1 kcal/mol. A production run of 100ps was then performed and the conformation of the final frame was saved as the relaxed conformation.

3 Optimization of P1LnkP2 conformations along the RDCs

The 33 selected conformations of P1LnkP2 displaying a R correlation factor smaller than 0.7 between experimental RDCs and RDCs calculated by PALES 2.1[9] were optimized along RDCs using XPLOR-NIH 3.1[10] script refine.py. This script, available as example in the XPLOR-NIH package, implements a slow cooling protocol in torsion angle space. The high

temperature of this protocol was modified to be set to 300K, as only a simple optimization of the structure to fit the RDC measurements was sought. The final temperature of the system was 25K. The RDC refinement was performed using the residual dipolar couplings (RDC) measured previously[1] on the NH groups of residues 137-222 (P1) and 281-374 (P2), using the axial and rhomboic components determined by PALES on each initial P1LnkP2 conformation. Restraints on ϕ and ψ backbone dihedral angles were applied on residues of P1 and P2 detected by STRIDE[11] as α-helix or β-strand in the starting P1LnkP2 conformation. The target values of the ϕ, ψ restraints were the values observed in the starting P1LnkP2 conformation, and a interval of 20° was allowed around the target value. Twenty P1LnkP2 conformations were produced by the refinement procedure, from which the conformation displaying the smallest total energy was selected.

4 Optimization of P1LnkP2 conformations along the PREs

The refinements of the 10 conformations (13, 20, 55, 145, 146, 150, 153, 160, 174, 186) displaying a distance between geometric centers of P1 and P2 smaller than 32 Å, with respect to the measured paramagnetic enhancements (PRE) were performed with the same script refine.py and conditions than the ones previously used for RDC refinement. In addition to RDCs and ϕ, ψ restraints, the volume of P1LnkP2 was restrained to 3500 Å^3 using the potential gyrPot.[12]

5 Determination of the P1LnkP2 populations

The relative populations of the 83 P1LnkP2 conformations were determined by fitting the SAXS curve previously measured.[1] Two software were used in parallel: BioEn 0.1.1,[13] based on optimization in a Bayesian frame, and Mesmer 1.0.0,[14] based on a genetic algorithm. On

each considered conformation, theoretical SAXS curves were calculated using CRYSOL[15] available in the package ATSAS 3.0.3[16] with 857 points, a maximum scattering vector of 4 nm^{-1} and a maximum order of harmonics of 18. A 1D cubic interpolation[17] was used to obtained the theoretical SAXS values at the same sets of scattering vectors q than the ones at which the experimental SAXS curve was recorded.

The processing with BioEn was performed in the following way. The optimization was run for 1000 steps using the GSL library.[18] Ten runs were performed independently on all considered conformations, and the subset of conformations for which the sum of observed populations larger than 0.01, was selected. Ten additional BioEn runs were performed on the subset of conformations, and from the results of the ten repetitions, average values and standard deviations were computed for the populations.

The genetic algorithm implemented in Mesmer starts the calculation with an initial set of conformations (called components) randomly chosen from the available pool of components.[14] Two sets of Mesmer calculations were performed, starting from either 40 or 60 initial components. Each type of run was repeated four times. The populations of conformations were then averaged and their standard deviations calculated.

6 Calculation of the backbone angles from interatomic distances

In the following, the covalent bond angles between atoms A, B and C are denoted by $ab(A, B, C)$, and the covalent bond length between atoms A and B by $b(A, B)$. An angle between three atoms A, B and C not connected by covalent bonds is denoted by $a(A, B, C)$ and the distance between atoms A and B not connected by a covalent bond is denoted by $d(A, B)$.

6.1 Calculation of backbone angle ϕ using covalent geometry along with the distance between carbonyls of two successive residues

The dihedral angle ϕ^i between atoms C^{i-1}, N^i, $\mathrm{C}\alpha^i$ and C^i, i being the residue number, is defined using the trihedron cosine law as:

$$\cos(\phi^i) = \frac{\cos\gamma - (\cos\alpha\cos\beta)}{\sin\alpha\sin\beta} \tag{1}$$

where the angles α, β and γ are respectively the angles between atoms (C^{i-1}, N^i, $\mathrm{C}\alpha^i$), ($\mathrm{C}\alpha^i$, N^i, C^i) and (C^{i-1}, N^i, C^i). The angle α is defined by the force field as the atoms C^{i-1}, N^i and $\mathrm{C}\alpha^i$ are connected by two covalent bonds:

$$\alpha = ab(C^{i-1}, N^i, C\alpha^i) \tag{2}$$

As, due to the protein stereo-chemistry, α is in the 0-180° range, $\sin\alpha = \sqrt{1-\cos^2\alpha}$.

Using the cosine law between atoms $\mathrm{C}\alpha^i$, N^i and C^i, $\cos\beta$ can be expressed as:

$$\cos(\beta) = -\frac{b^2(C\alpha^i, C^i) - b^2(C\alpha^i, N^i) - d^2(C^i, N^i)}{2b(N^i, C\alpha^i)d(N^i, C^i)} \tag{3}$$

Due to the protein stereo-chemistry, β is in the 0-180° range and $\sin\beta = \sqrt{1-\cos^2\beta}$. Using the cosine law between atoms C^{i-1}, N^i and C^i, $\cos\gamma$ can be expressed as;

$$\cos(\gamma) = -\frac{d^2(C^{i-1}, C^i) - b^2(C^{i-1}, N^i) - d^2(N^i, C^i)}{2b(N^i, C^{i-1})d(N^i, C^i)} \tag{4}$$

The distance $d(N^i, C^i)$, which is the only parameter not defined by the force field in Eqs. 3 and 4 can be calculated using another cosine law expression between atoms N^i, $\mathrm{C}\alpha^i$ and C^i:

$$d^2(N^i, C^i) = b^2(N^i, C\alpha^i) + b^2(C\alpha^i, C^i) - 2b(C\alpha^i, N^i)b(C\alpha^i, C^i)\cos(\delta) \tag{5}$$

where the angle δ is obtained from the force field: $\delta = ab(N^i, C\alpha^i, C^i)$.

6.2 Calculation of the backbone angle ψ using covalent geometry along with the distance between the nitrogens of two successive residues

The dihedral angle ψ^i between atoms N^i, $C\alpha^i$, C^i and N^{i+1}, i being the residue number, is defined using the trihedron cosine law as:

$$\cos(\psi^i) = \frac{\cos\gamma - (\cos\alpha\cos\beta)}{\sin\alpha\sin\beta} \tag{6}$$

where the angles α, β and γ are defined as the angles between atoms (N^i, $C\alpha^i$, C^i), (C^i, $C\alpha^i$, N^{i+1}) and (N^i, $C\alpha^i$, N^{i+1}). The angle α is defined by the force field as the atoms N^i, $C\alpha^i$ and C^i are connected by two covalent bonds:

$$\alpha = ab(N^i, C\alpha^i, C^i) \tag{7}$$

and because of the protein stereo-chemistry, α is in the 0-180° range and $\sin\alpha = \sqrt{1-\cos^2\alpha}$.

Using the cosine law between atoms C^i, $C\alpha^i$ and N^{i+1}, $\cos\beta$ can be expressed as:

$$\cos(\beta) = -\frac{b^2(C^i, N^{i+1}) - b^2(C^i, C\alpha^i) - d^2(C\alpha^i, N^{i+1})}{2b(C\alpha^i, C^i)d(C\alpha^i, N^{i+1})} \tag{8}$$

Due to the protein stereo-chemistry, β is in the 0-180° range and $\sin\beta = \sqrt{1-\cos^2\beta}$. Using the cosine law between the atoms N^i, $C\alpha^i$ and N^{i+1}, $\cos\gamma$ can be expressed as;

$$\cos(\gamma) = -\frac{d^2(N^i, N^{i+1}) - b^2(N^i, C\alpha^i) - d^2(C\alpha^i, N^{i+1})}{2b(C\alpha^i, N^i)d(C\alpha^i, N^{i+1})} \tag{9}$$

The distance $d(C\alpha^i, N^{i+1})$, which is the only parameter not defined by the force field in Eqs. 8 and 9 can be calculated using another cosine law expression between atoms $C\alpha^i$, C^i

and N^{i+1}:

$$d^2(C\alpha^i, N^{i+1}) = b^2(C\alpha^i, C^i) + b^2(C^i, N^{i+1}) - 2b(C^i, C\alpha^i)b(C^i, N^{i+1})\cos(\delta) \qquad (10)$$

where the angle δ is obtained from the force field: $\delta = ab(C\alpha^i, C^i, N^{i+1})$.

Table S1. see caption on the next page.

Residue	ϕ intervals	ψ intervals	RCI S^2	Residue	ϕ intervals	ψ intervals	RCI S^2
Y-220	-180.0 -100.0	60.0 160.0	0.870	Q-241	-140.0 -40.0	-80.0 20.0	0.451
**S-221**	-180.0 -60.0	60.0 160.0	0.768	**G-242**	-100.0 -80.0	140.0 160.0	0.300
A-222	-140.0 -20.0	-80.0 20.0	0.486	**G-242**	40.0 100.0	-40.0 40.0	
A-222	-100.0 -40.0	80.0 160.0		**G-242**	-100.0 -40.0	-80.0 0.0	
A-222	-100.0 -40.0	80.0 160.0		R-243	-140.0 -40.0	-80.0 20.0	0.274
**G-223**	-180.0 -100	120.0 180.0	0.409	R-243	-120.0 -60.0	80.0 160.0	
**G-223**	-120.0 -40.0	-80.0 0.0		**S-244**	-140.0 -40.0	-80.0 20.0	0.333
**G-223**	60.0 80.0	-20.0 20.0		**S-244**	-140.0 -60.0	80.0 180.0	
**G-223**	100.0 160.0	120.0 180.0		T-245	-160.0 -80.0	80.0 180.0	0.487
R-224	-140.0 -60.0	-80.0 20.0	0.347	T-245	-140.0 -60.0	-60.0 40.0	
R-224	-140.0 -60.0	80.0 160.0		**S-246**	-180.0 -80.0	40.0 180.0	0.600
**I-225**	-160.0 -60.0	40.0 160.0	0.410	P-247	-160.0 -140.0	140.0 160.0	0.611
P-226	-120.0 -40.0	60.0 180.0	0.395	P-247	-100.0 -80.0	-80.0 -20.0	
P-226	-100.0 -60.0	-60.0 -20.0		P-247	-100.0 -20.0	80.0 180.0	
**G-227**	-120.0 -40.0	-60.0 40.0	0.395	**P-248**	-160.0 -20.0	80.0 180.0	0.456
**G-227**	40.0 100.0	-60.0 40.0		**P-248**	-100.0 -80.0	-60.0 -20.0	
G-228	-140.0 -40.0	120.0 180.0	0.382	S-249	-140.0 -40.0	-60.0 20.0	0.392
G-228	-120.0 -40.0	-80.0 0.0		S-249	-120.0 -40.0	80.0 180.0	
G-228	60.0 100.0	-40.0 20.0		**S-250**	-160.0 -60.0	100.0 160.0	0.305
**Y-229**	-180.0 -60.0	80.0 180.0	0.345	**S-250**	-140.0 -40.0	-60.0 40.0	
**Y-229**	-120.0 -80.0	-60.0 0.0		L-251	-140.0 -40.0	60.0 160.0	0.275
V-230	-160.0 -60.0	80.0 180.0	0.333	**P-252**	-120.0 -40.0	60.0 180.0	0.261
V-230	-120.0 -60.0	-60.0 0.0		**P-252**	-100.0 -40.0	-80.0 -20.0	
**T-231**	-140.0 -40.0	-80.0 20.0	0.381	Q-253	-100.0 -80.0	-60.0 -20.0	-
**T-231**	-120.0 -60.0	80.0 180.0		Q-253	-120.0 -40.0	80.0 180.0	
N-232	-140.0 -40.0	-80.0 20.0	0.448	**P-254**	-120.0 -60.0	-60.0 20.0	0.508
N-232	-120.0 -60.0	80.0 160.0		**P-254**	-120.0 -40.0	80.0 180.0	-
**H-233**	-140.0 -40.0	-80.0 20.0	0.516	H-255	-120.0 -60.0	-60.0 20.0	0.474
**H-233**	-120.0 -60.0	80.0 160.0		H-255	-120.0 -40.0	80.0 180.0	
I-234	-160.0 -60.0	80.0 160.0	0.567	**G-256**	-120.0 -60.0	-60.0 0.0	0.447
I-234	-120.0 -40.0	-80.0 0.0		**G-256**	40.0 80.0	-180.0 -100.0	
**Y-235**	-160.0 -60.0	60.0 160.0	0.648	**G-256**	-100.0 -40.0	80.0 160.0	
**Y-235**	-100.0 -60.0	-60.0 0.0		S-257	-120.0 -20.0	-80.0 20.0	0.387
T-236	-160.0 -60.0	60.0 160.0	0.733	S-257	-100.0 -40.0	80.0 180.0	
T-236	-100.0 -40.0	-100.0 -20.0		**T-258**	-140.0 -60.0	-80.0 20.0	0.323
**W-237**	-160.0 -60.0	80.0 160.0	0.724	**T-258**	-120.0 -60.0	100.0 160.0	
**W-237**	-120.0 -40.0	-100.0 0.0		L-259	-120.0 -40.0	-100.0 0.0	0.254
V-238	-160.0 -60.0	80.0 160.0	0.732	L-259	-100.0 -40.0	100.0 160.0	
V-238	-120.0 -40.0	-80.0 20.0		**R-260**	-120.0 -40.0	-100.0 0.0	0.211
**D-239**	-160.0 -40.0	40.0 160.0	0.734	**R-260**	-100.0 -40.0	100.0 160.0	
P-240	-100.0 -40.0	-100.0 0.0	0.607	Q-261	-140.0 -40.0	-100.0 20.0	0.258
P-240	-100.0 -40.0	100.0 160.0		Q-261	-120.0 -40.0	100.0 160.0	

Table S1. List of ϕ, ψ restraint boxes and of the random coil index (RCI) order parameter S^2 [19] calculated by TALOS-N [20] along the Lnk residues. Each line contains the definition of one restraint box. Several restraint boxes were determined for a given residue.

Residue	ϕ intervals	ψ intervals	RCI S^2	Residue	ϕ intervals	ψ intervals	RCI S^2
R-262	-100.0 -40.0	100.0 160.0	0.389	L-272	-120.0 -40.0	-100.0 0.0	0.309
R-262	-120.0 -40.0	-100.0 0.0		L-272	-120.0 -40.0	80.0 160.0	
**E-263**	-120.0 -40.0	-100.0 0.0	0.613	**L-273**	-120.0 -40.0	-100.0 0.0	0.221
**E-263**	-100.0 -40.0	100.0 160.0		**L-273**	-120.0 -40.0	80.0 160.0	
D-264	-120.0 -40.0	-100.0 20.0	0.762	Q-274	-140.0 -40.0	-80.0 20.0	0.182
**D-265**	-120.0 -40.0	-100.0 20.0	0.787	Q-274	-120.0 -60.0	80.0 160.0	
R-266	-120.0-40.0	-100.0 0.0	0.718	**S-275**	-140.0 -40.0	-80.0 20.0	0.242
R-266	-80.0 -60.0	100.0 140.0		**S-275**	-120.0 -40.0	80.0 180.0	
**R-267**	-120.0 -40.0	-100.0 0.0	0.653	G-276	-140.0 -60.0	120.0 160.0	0.401
S-268	-140.0 -40.0	-100.0 20.0	0.557	G-276	-120.0 -20.0	-80.0 0.0	
S-268	-80.0 -60.0	100.0 160.0		G-276	60.0 100.0	-40.0 20.0	
**T-269**	-140.0 -40.0	-100.0 20.0	0.492	**D-277**	-140.0 -40.0	-80.0 20.0	0.631
**T-269**	-100.0 -60.0	120.0 160.0		**D-277**	-100.0 -60.0	100.0 160.0	
L-270	-120.0 -40.0	-100.0 0.0	0.422	E-278	-180.0 -60.0	80.0 180.0	0.735
L-270	-100.0 -60.0	100.0 140.0		E-278	-120.0 -80.0	-60.0 0.0	
**H-271**	-140.0 -60.0	-100.0 20.0	0.371	**K-279**	-180.0 -60.0	80.0 180.0	0.802
**H-271**	-120.0 -60.0	80.0 160.0		K-280	-160.0 -60.0	60.0 160.0	0.830

Table S2. Populations of conformations found using Mesmer 1.0.0.[14] Two sets of calculation were run using initial sets of 40 or 60 conformations (components). The final scores for the two runs are equal to 1.332.

	40 components	60 components
conf12	19.9 ± 1.9	20.1 ± 1.5
conf24	6.9 ± 0.54	7.1 ± 0.59
conf140	10.5 ± 0.11	10.3 ± 0.47
conf150	12.8 ± 0.19	12.8 ± 0.18
conf176	49.8 ± 1.6	49.4 ± 1.1

Table S3. RMS, R and Q factors calculated between experimental PRE profiles and theoretical PRE profile calculated on each selected closed conformation. For each closed conformations, the parameters are given for each MSTL spin label.

conf13	Spin label	RMS	R	Q factor
	162	0.31	0.57	0.43
	212	0.23	0.72	0.29
	231	0.38	0.23	0.62
	290	0.31	0.63	0.48
	371	0.29	0.62	0.51
conf20	Spin label	RMS	R	Q factor
	162	0.29	0.62	0.41
	212	0.20	0.78	0.25
	231	0.43	0.01	0.69
	290	0.31	0.53	0.49
	371	0.32	0.52	0.56
conf55	Spin label	RMS	R	Q factor
	162	0.34	0.37	0.47
	212	0.22	0.73	0.27
	231	0.40	0.15	0.65
	290	0.32	0.59	0.51
	371	0.41	0.33	0.73

Table S4. Q factor, RMS (Hz) and R correlation factor calculated between experimental and calculated RDCs for each conformation shown in Figure 3 of the main text. The conformations belonging to the set of closed conformations are written in bold.

	RDC Q factor	RDC RMS	RDC R correlation
conf12	0.675	5.346	0.746
**conf13**	**0.160**	**1.268**	**0.987**
**conf20**	**0.119**	**0.940**	**0.993**
conf24	0.692	5.478	0.725
**conf55**	**0.342**	**2.710**	**0.942**
conf140	0.157	1.242	0.988
conf176	0.581	4.601	0.817

Table S5. Atom re-ordering used during the iBP calculation step within the first, the last and the inner residues of the peptide fragment. The order is described by the list of atoms names, the signs "-" and "+" describing atoms located in the previous and the next residues in the primary sequence.

Residue position	order
first	N, H1, H2, CA, N, HA, CA, C
inner	N, -O, -CA, -C, N, CA, C, +N,<br>-C, N, CA, H1, N, CA, C, HA, C, CA
last	N, -O, -CA, -C, N, CA, C,<br>-C, N, CA, H1, N, CA, C, HA,<br>C, CA, O, C, O2

Table S6. Atom types and geometric parameters for covalent and improper bonds and bond angles taken from the force field PARALLHDG (version 5.3).[21]

Atom name	Definition of atom type	Bond atoms	Bond length (Å)
CH1E	α carbon	C-CH1E	1.525
C	carbonyl carbon	CH1E-HA	1.080
O	carbonyl oxygen	CH1E-NH1	1.458
OC	C-terminal carbonyl oxygen	CH1E-NH2	1.486
HA	Hα hydrogen	C-NH1	1.329
NH1	amide nitrogen	C-O	1.231
NH2	N-terminal amide	C-OC	1.249
	nitrogen	H-NH1	0.980
H	amide hydrogen	H-NH2	0.980
**Bond angle atoms**	**Bond angle value (°)**	**Improper angle atoms**	**Improper angle value (°)**
C-CH1E-HA	108.9914	C-CH1E-HA-HA	-70.4072
C-CH1E-NH1	111.1396	CH1E-C-NH1-HA	66.2535
C-CH1E-NH2	106.9610	C-NH1-HA-HA	-70.8745
C-NH1-CH1E	121.6541	HA-CH1E-HA-HA	-66.5692
CH1E-C-NH1	116.1998	CH1E-C-NH1-CH1E	178.0
CH1E-C-O	120.8258	O-C-NH1-H	178.0
CH1E-NH1-H	119.2367	C-CH1E-NH1-O	0.0
C-NH1-H	119.2489	NH1-CH1E-C-HA	119.0
C-NH2-H	118.1853	NH2-CH1E-C-HA	119.0
HA-CH1E-NH1	108.0508	NH1-HA-CH1E-C	121.0
H-NH2-CH1E	109.5000	NH2-HA-CH1E-C	116.0
H-NH2-H	107.3000	C-NH1-CH1E-H	180.0
NH1-C-O	122.9907	CH1E-C-NH1-O	180.0
NH2-CH1E-HA	108.4800	NH2-H-H-CH1E	41.0
NH2-C-O	122.6277	CH1E-OC-C-OC	178.0
CH1E-C-OC	118.0611		
OC-C-OC	123.3548		

Table S7. Population results obtained using only the SAXS data measured for scattering vectors q up to 3.5 nm^{-1}. Populations of conformations found using BioEn 0.1.1[13] on various sets of conformations including the 73 P1LnkP2 conformations for which the distance between the geometric centers of P1 and P2 was larger than 32 Å and various closed conformations among 13, 20, 55, 150, previously selected according to the fit of PRE data. After ten runs starting from random values of populations and performed on the whole set of conformations, all conformations for which the sum of populations over the ten runs was larger than 0.01 were gathered, and ten additional BioEn calculations were performed on this reduced set of conformations. The average and standard deviation values of populations obtained for each selected conformation from the second set of BioEn runs, are given in the Table, along with the final average values of χ^2 and of entropy S. In each calculation, the conformations tagged as "not incl" have not been initially included in the calculation, whereas the conformations tagged as "-" were included in the calculation, but not selected by BioEn.[13] The conformations belonging to the set of closed conformations are written in bold.

	conf13	conf20	conf55	conf13,20,55	conf13,20,55,150
conf12	2.2 ± 0.75	-	1.5 ± 0.75	4.2 ± 3.3e-3	72 ± 0.84
**conf13**	**9.8 ± 0.07**	**not incl**	**not incl**	**-**	**-**
**conf20**	**not incl**	**9.0 ± 4.3e-4**	**not incl**	**-**	**3.1 ± 6.1**
conf24	15 ± 0.24	19 ± 2.1e-3	19 ± 6.1	16 ± 1.1e-3	-
**conf55**	**not incl**	**not incl**	**9.9 ± 1.1**	**10 ± 4.0e-6**	**13 ± 6.7**
conf71	-	-	0.3 ± 0.4		
conf140	8.6 ± 0.029	8.1 ± 6.2e-4	7.8 ± 2.6	8.8 ± 3.8e-4	11 ± 0.3
**conf150**	**not incl**	**not incl**	**not incl**	**not incl**	**-**
conf176	64 ± 0.61	64 ± 2.3e-3	61 ± 1.8	60 ± 2.8e-3	-
Final $\chi 2$	1.6 ± 5.1e-7	5.0 ± 1.6e-6	2.5 ± 9.1e-7	2.2 ± 3.6e-7	6.0 ± 1.3e-6
Final S	-1.9e-5 ± 4.4e-10	-8.7e-5 ± 1.4e-9	-4.1e-5 ± 7.7e-10	-1.9e-5 ± 3.0e-10	-5.5e ± 1.1e-9

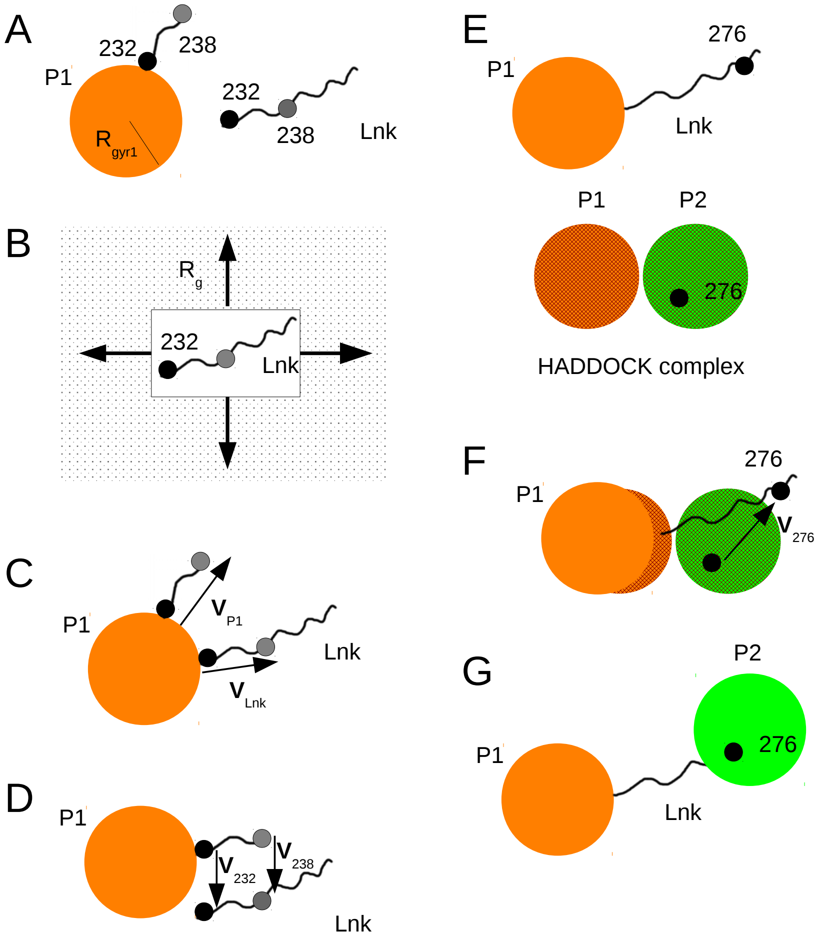

Figure S1. Scheme for assembling P1 and P2 domains to the Lnk conformations. A. Initial P1 and Lnk conformations. B. Definition of the grid for translating the P1 geometric center. C. Rotation of translated P1 to superimpose vectors $\mathbf{V}_{P1}$ and $\mathbf{V}_{Lnk}$. D. translation of P1 with respect of vectors $\mathbf{V}_{232}$ and $\mathbf{V}_{238}$. E. P1Lnk conformation determined from the stages A-D and P1P2 complex predicted using HADDOCK.[22] F. Superimposition of P1 conformations in P1Lnk and in the HADDOCK P1P2 complex and definition of the translation vector $\mathbf{V}_{276}$. G. P1LnkP2 conformation with translated P2 according to the vector $\mathbf{V}_{276}$. This scheme was prepared using LibreOffice 6.0.7.3.[23]

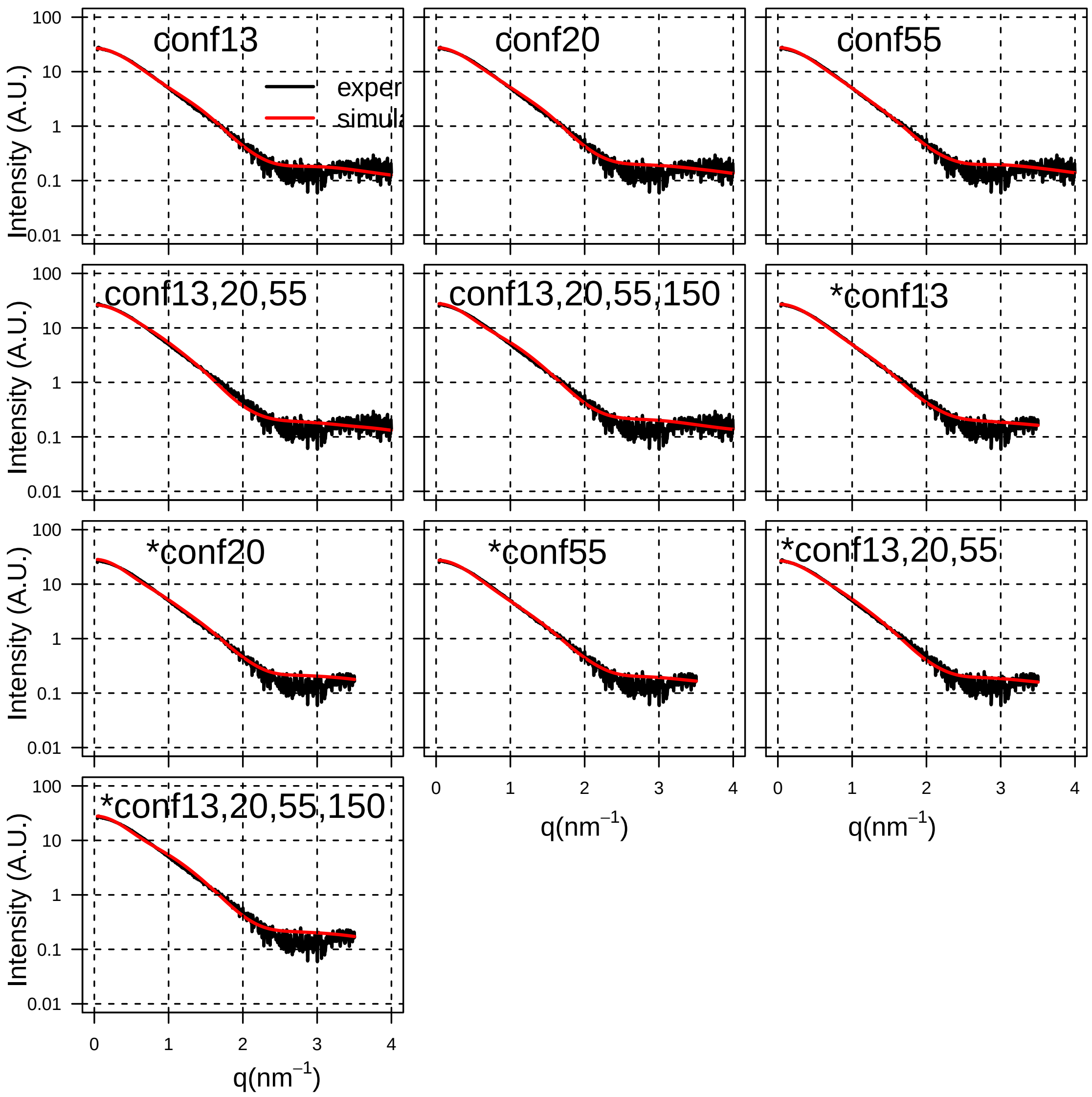

Figure S2. Superimposition of experimental (black curves) and theoretical (red curves) SAXS data for representative calculations of populations for each of the runs indicated in Table 2 and S7. The runs described in Table S7 and based on the SAXS data measured for scattering vectors q up to only 3.5 nm^{-1} are labeled with stars. The plots were prepared using R 3.4.1.[24]

Figure S3. **On the two next pages**. Superimposition of experimental (M_{obs}, black contours) and theoretical (M_{theo}, red contours) likelihood maps obtained by TALOS-N for each residue of Lnk. For M_{obs}, the TALOS-N inputs were the experimental chemical shifts whereas for M_{theo}, the inputs were the chemical shifts calculated on the conformations 12, 13, 24, 140, 176 and averaged according to the populations displayed in Figure 3 in the main text. The plots were prepared using R 3.4.1.[24]

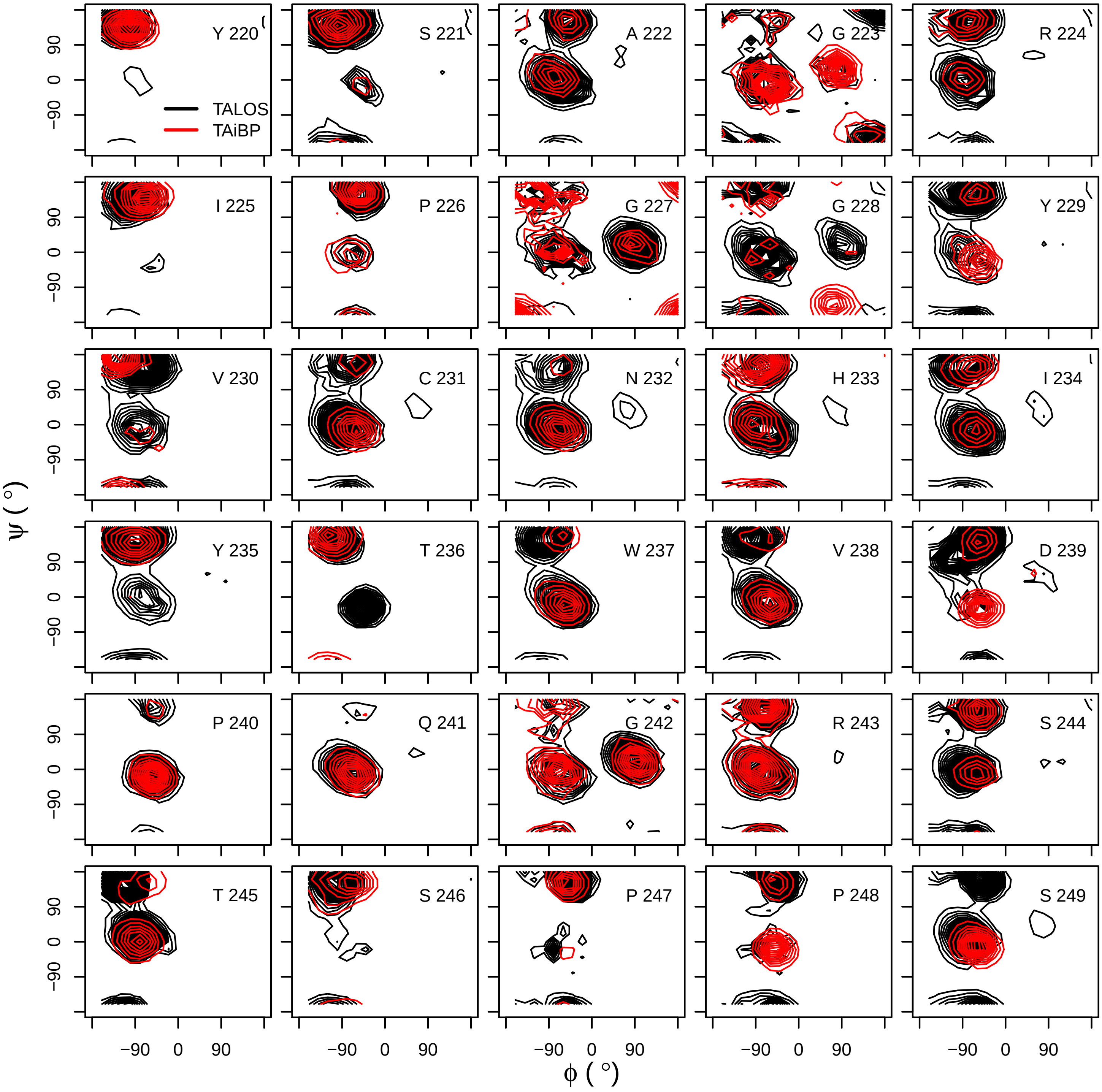

Y 220
S 221
A 222
G 223
R 224
I 225
P 226
G 227
G 228
Y 229
V 230
C 231
N 232
H 233
I 234
Y 235
T 236
W 237
V 238
D 239
P 240
Q 241
G 242
R 243
S 244
T 245
S 246
P 247
P 248
S 249
TALOS
TAiBP
ψ (°)
φ (°)
−90
0
90

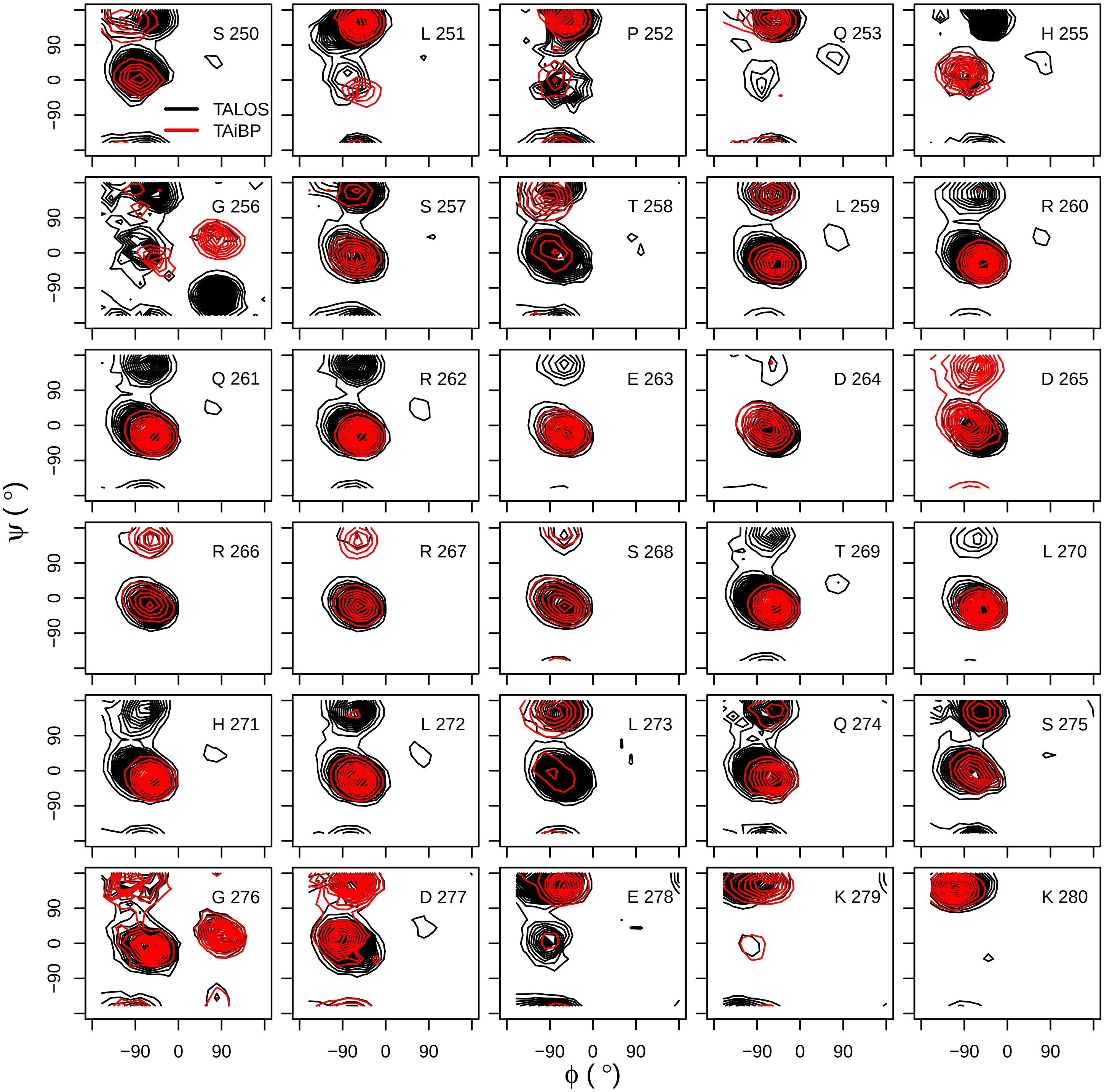

S 250
L 251
P 252
Q 253
H 255
G 256
S 257
T 258
L 259
R 260
Q 261
R 262
E 263
D 264
D 265
R 266
R 267
S 268
T 269
L 270
H 271
L 272
L 273
Q 274
S 275
G 276
D 277
E 278
K 279
K 280
TALOS
TAiBP
ψ (°)
ϕ (°)
−90
0
90

References

(1) Delhommel, F.; Cordier, F.; Bardiaux, B.; Bouvier, G.; Colcombet-Cazenave, B.; Brier, S.; Raynal, B.; Nouaille, S.; Bahloul, A.; Chamot-Rooke, J.; Nilges, M.; Petit, C.; Wolff, N. Structural Characterization of Whirlin Reveals an Unexpected and Dynamic Supramodule Conformation of Its PDZ Tandem. *Structure* **2017**, *25*, 1645–1656.

(2) Phillips, J.; Braun, R.; Wang, W.; Gumbart, J.; Tajkhorshid, E.; Villa, E.; Chipot, C.; Skeel, R.; Kale, L.; Schulten, K. Scalable molecular dynamics with NAMD. *J Comput Chem* **2005**, *26*, 1781–1802.

(3) Best, R. B.; Zhu, X.; Shim, J.; Lopes, P. E.; Mittal, J.; Feig, M.; Mackerell, A. D. Optimization of the additive CHARMM all-atom protein force field targeting improved sampling of the backbone ϕ and ψ and side-chain $\chi(1)$ and $\chi(2)$ dihedral angles. *J Chem Theory Comput* **2012**, *8*, 3257–3273.

(4) Huang, J.; Rauscher, S.; Nawrocki, G.; Ran, T.; Feig, M.; de Groot, B. L.; Grubmller, H.; MacKerell, A. D. CHARMM36m: an improved force field for folded and intrinsically disordered proteins. *Nat Methods* **2017**, *14*, 71–73.

(5) Tanner, D. E.; Chan, K. Y.; Phillips, J. C.; Schulten, K. Parallel Generalized Born Implicit Solvent Calculations with NAMD. *J Chem Theory Comput* **2011**, *7*, 3635–3642.

(6) Ryckaert, J.; Ciccotti, G.; Berendsen, H. Numerical integration of the cartesian equations of motion of a system with constraints and Molecular dynamics of n-alkanes. *J. Comput. Phys.* **1977**, *23*, 327–341.

(7) Andersen, H. Rattle: a "Velocity" Version of the Shake Algorithm for Molecular Dynamics Calculations. *J Comp Phys* **1983**, *52*, 24–34.

(8) Frenkel, D.; Smit, B. *Understanding molecular simulation: from algorithms to applications*; Academic press: San Diego, California, 2002.

(9) Zweckstetter, M.; Bax, A. Prediction of sterically induced alignment in a dilute liquid crystalline phase: aid to protein structure determination by NMR. *J Am Chem Soc* **2000**, *122*, 3791–3792.

(10) Schwieters, C. D.; Bermejo, G. A.; Clore, G. M. Xplor-NIH for molecular structure determination from NMR and other data sources. *Protein Sci* **2018**, *27*, 26–40.

(11) Frishman, D.; Argos, P. Knowledge-based protein secondary structure assignment. *Proteins* **1995**, *23*, 566–579.

(12) Schwieters, C. D.; Clore, G. M. A pseudopotential for improving the packing of ellipsoidal protein structures determined from NMR data. *J Phys Chem B* **2008**, *112*, 6070–6073.

(13) Kfinger, J.; Stelzl, L. S.; Reuter, K.; Allande, C.; Reichel, K.; Hummer, G. Efficient Ensemble Refinement by Reweighting. *J Chem Theory Comput* **2019**, *15*, 3390–3401.

(14) Ihms, E. C.; Foster, M. P. MESMER: minimal ensemble solutions to multiple experimental restraints. *Bioinformatics* **2015**, *31*, 1951–1958.

(15) Svergun, D. I.; Barberato, C.; Koch, M. CRYSOL - a Program to Evaluate X-ray Solution Scattering of Biological Macromolecules from Atomic Coordinates. *J. Appl. Cryst.* **1995**, *28*, 768–773.

(16) Manalastas-Cantos, K.; Konarev, P. V.; Hajizadeh, N. R.; Kikhney, A. G.; Petoukhov, M. V.; Molodenskiy, D. S.; Panjkovich, A.; Mertens, H. D. T.; Gruzinov, A.; Borges, C.; Jeffries, C. M.; Svergun, D. I.; Franke, D. ATSAS 3.0: expanded functionality and new tools for small-angle scattering data analysis. *J Appl Crystallogr* **2021**, *54*, 343–355.

(17) Virtanen, P. et al. SciPy 1.0: Fundamental Algorithms for Scientific Computing in Python. *Nature Methods* **2020**, *17*, 261–272.

(18) Galassi, M. *GNU Scientific Library Reference Manual (3rd Ed.)”*.

(19) Berjanskii, M. V.; Wishart, D. S. A simple method to predict protein flexibility using secondary chemical shifts. *J Am Chem Soc* **2005**, *127*, 14970–14971.

(20) Shen, Y.; Bax, A. Protein structural information derived from NMR chemical shift with the neural network program TALOS-N. *Methods Mol Biol* **2015**, *1260*, 17–32.

(21) Engh, R.; Huber, R. Accurate bond and angle parameters for X-ray protein structure refinement. *Acta Crystallogr A* **1991**, *47*, 392–400.

(22) Saponaro, A.; Maione, V.; Bonvin, A. M. J. J.; Cantini, F. Understanding Docking Complexes of Macromolecules Using HADDOCK: The Synergy between Experimental Data and Computations. *Bio Protoc* **2020**, *10*, e3793.

(23) LibreOffice Community, LibreOffice. The Document Foundation.

(24) R Core Team, R: A Language and Environment for Statistical Computing. R Foundation for Statistical Computing: Vienna, Austria, 2017.
